# Genetic basis for plasma amino acid concentrations based on absolute quantification: a genome-wide association study in the Japanese population

**DOI:** 10.1038/s41431-018-0296-y

**Published:** 2019-01-18

**Authors:** Akira Imaizumi, Yusuke Adachi, Takahisa Kawaguchi, Koichiro Higasa, Yasuharu Tabara, Kazuhiro Sonomura, Taka-aki Sato, Meiko Takahashi, Toshimi Mizukoshi, Hiro-o Yoshida, Naoko Kageyama, Chisato Okamoto, Mariko Takasu, Maiko Mori, Yasushi Noguchi, Nobuhisa Shimba, Hiroshi Miyano, Ryo Yamada, Fumihiko Matsuda

**Affiliations:** 1 Institute for Innovation, Ajinomoto Co., Inc., Kawasaki, Kanagawa 210-8681 Japan; 2 Center for Genomic Medicine, Kyoto University Graduate School of Medicine, Kyoto, 606-8507 Japan; 3 Department of Genome Analysis, Institute of Biomedical Science, Kansai Medical University, Hirakata, Osaka, 573-1010 Japan; 4 Life Science Laboratories, Shimadzu Corporation, Seika, Kyoto 619-0237 Japan; 5 R&D Planning Dept., Ajinomoto Co., Inc, Tokyo, 104-8315 Japan

**Keywords:** Genome-wide association studies, Metabolomics

## Abstract

To assess the use of plasma free amino acids (PFAAs) as biomarkers for metabolic disorders, it is essential to identify genetic factors that influence PFAA concentrations. PFAA concentrations were absolutely quantified by liquid chromatography–mass spectrometry using plasma samples from 1338 Japanese individuals, and genome-wide quantitative trait locus (QTL) analysis was performed for the concentrations of 21 PFAAs. We next conducted a conditional QTL analysis using the concentration of each PFAA adjusted by the other 20 PFAAs as covariates to elucidate genetic determinants that influence PFAA concentrations. We identified eight genes that showed a significant association with PFAA concentrations, of which two, *SLC7A2* and *PKD1L2*, were identified. *SLC7A2* was associated with the plasma levels of arginine and ornithine, and *PKD1L2* with the level of glycine. The significant associations of these two genes were revealed in the conditional QTL analysis, but a significant association between serine and the *CPS1* gene disappeared when glycine was used as a covariate. We demonstrated that conditional QTL analysis is useful for determining the metabolic pathways predominantly used for PFAA metabolism. Our findings will help elucidate the physiological roles of genetic components that control the metabolism of amino acids.

## Introduction

Circulating metabolite concentrations in the body can serve as useful biomarkers for the diagnosis, prognosis, and risk assessment of diseases, particularly for metabolic disorders such as diabetes, dyslipidemia, and hypertension [1–8]. Among these metabolites, the free amino acids in plasma (PFAAs) are key regulators of metabolic pathways, and their concentrations are influenced by both genetic and environmental factors, such as the diet [9–14].

Recently, several genome-wide association studies (GWAS) also identified genetic variations associated with PFAAs in European populations [9–11, 13, 15]. However, the influence of heritability and whether these loci are shared among other human populations are still unknown. In addition, metabolite concentrations are influenced by other metabolites within the same metabolic pathway. Therefore, genome-wide quantitative trait locus (QTL) analyses conditioned on the other amino acids sharing the same pathway are necessary.

In this study, we sought to elucidate genetic determinants that influence PFAA concentrations. We conducted a QTL analysis of PFAAs measured by an absolute quantification method using plasma samples from 1338 Japanese individuals.

## Materials and methods

### Subjects and ethics

Participants were recruited from the Nagahama Prospective Genome Cohort for Comprehensive Human Bioscience (the Nagahama Study). All of the subjects were approved by the Institutional Review Board and the ethics committees of each institute, to which donors gave written informed consent, in accordance with the national guidelines.

### Absolute quantification of PFAA concentrations

The concentrations of 21 PFAAs from 2,084 individuals who participated in the Nagahama study in 2008 (*n* = 1124) and 2009 (*n* = 960) were quantified. Blood samples (5 ml) were collected from forearm veins after overnight fasting into tubes containing ethylenediaminetetraacetic acid (EDTA; Termo, Tokyo, Japan). The plasma was extracted by centrifugation at 2010×*g* at 4 °C for 15 min and then stored at –80 °C. After deproteinizing the thawed plasma samples using 80% acetonitrile, the samples were subjected to pre-column derivatization, then the absolute concentrations, the absolute concentrations of the PFAAs were measured by high performance liquid chromatography - electrospray ionization mass spectrometry (HPLC–ESI–MS). The methods were previously developed and verified by the authors [16–19].

Quantification was considered successful when the obtained value was within the determination range of the calibration curve.

### PFAA concentrations for QTL analysis

For the QTL analysis, we prepared three adjusted PFAA concentrations from the measured absolute concentrations of PFAAs. The first adjusted concentration was adjusted for sex and age by linear regression after Box-Cox transformation. The second was adjusted by the other 20 PFAAs by multiple linear regression after the first adjustment. For this regression analysis, explanatory variables were selected by the step-wise function (stepwiseglm) in MATLAB, with *P* = 0.001 and *P* *=* 0.01 as inclusion and exclusion criteria, respectively. The third was adjusted by one of the other PFAAs by linear regression after the first adjustment.

### SNP genotyping and quality control (QC) process

**Fig. 1:**
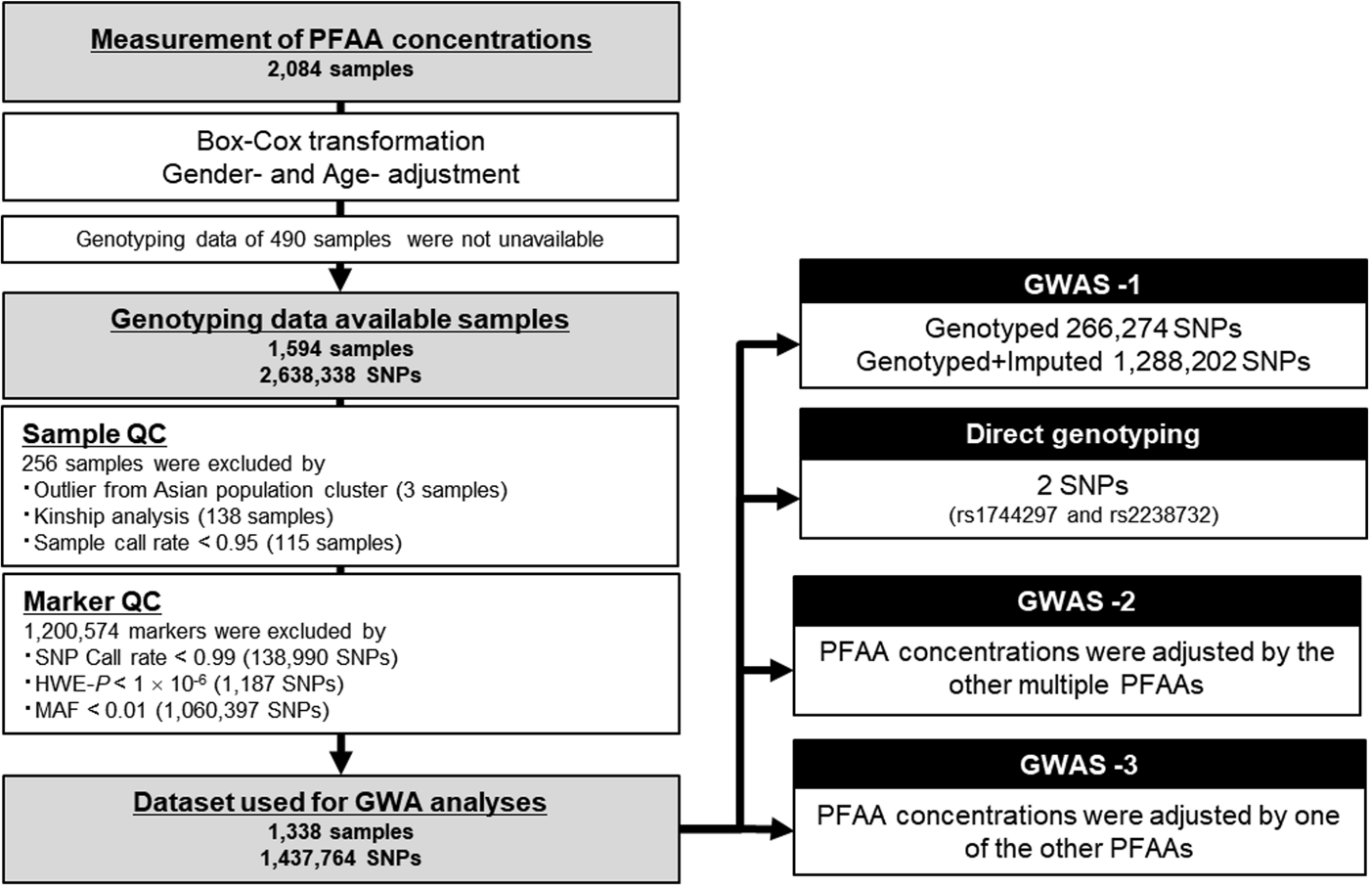
Flow diagram of the QC processes and QTL analyses using the PFAA concentrations of Japanese subjects from the Nagahama Study

A total of 1594 samples were genotyped using three commercially available Illumina genotyping arrays (Illumina, Inc., San Diego, CA): Human610-Quad BeadChip (610 K), HumanOmni-2.5-Quad BeadChip (2.5M-4), and HumanOmni-2.5-8 BeadChip (2.5M-8). The 1,124 subjects recruited in 2008 were genotyped using 610 K (*n* = 1,113) or both 610 K and 2.5M-4 (*n* = 11). The 470 subjects recruited in 2009 were genotyped using 610 K (*n* = 101), 2.5M-4 (*n* = 293), 2.5M-8 (*n* = 62), or both 610 K and 2.5M-4 (*n* = 14). In total, 2,638,338 SNPs were genotyped in the arrays. As shown in Fig. 1, through a sample QC process, 256 samples were excluded from the analysis: 3 genetic outliers identified by principal component analysis (PCA), 138 relatives, and 115 samples with a call rate of SNPs < 0.95. Through a marker QC process, 1,200,574 SNPs were excluded: 138,990 SNPs with a call rate < 0.99, 1,187 SNPs with deviation from Hardy–Weinberg equilibrium (*P* < 1.0 × 10^–6^), and 1,060,397 SNPs with a variant allele frequency < 1%. After the QC processes, 1,437,764 SNPs in 1338 samples remained for the GWA studies. Of these, 266,274 SNPs shared among all of the arrays were defined as intersectional SNPs. All of the QC procedures were processed using PLINK ver. 1.07 [20]. Both genotype and PFAA concentrations data of Nagahama study is deposited on the Japanese Genotype-phenotype Archive affiliated to the DDBJ (DNA Data Bank of Japan), via National Bioscience DataBase (NBDC), Japan. The data is accessible on hum0012 at https://ddbj.nig.ac.jp/jga/viewer/permit/dataset/JGAD00000000012.

### Imputation

The 1,437,764 SNPs from 1,338 samples used for the GWA studies were imputed using MACH ver. 1.0 [21]. An imputation panel was generated using the genotyping data of 665 Nagahama Study samples that were not used for the present GWA analyses and contained the results of 1,560,699 SNPs with Illumina HumanCoreExome BeadChip (Exome), HumanOmni2.5 S BeadChip (2.5 S), 2.5M-4, and 2.5M-8 arrays. Of these 665 samples, 478 were genotyped using all of the arrays, and 187 were genotyped using the Exome, 2.5 S, and 2.5M-4. Imputed SNPs with a variant allele frequency > 1% or an *r*^2^ < 0.5 were excluded from the subsequent association analysis. Finally, 1,288,202 SNPs from the 1338 samples were fixed with 1,021,918 additional SNPs.

### QTL analysis

For the three PFAA concentrations described above, QTL analysis was conducted with a an additive model implemented in PLINK [2.0]. The genome-wide significance threshold after Bonferroni correction was *P* < 3.88 × 10^–8^.

### Direct genotyping

The direct genotyping of two imputed SNPs (rs1744297 and rs2238732) was performed with TaqMan® SNP Genotyping Assays using the ABI PRISM 7700 system (Applied Biosystems, Foster City, CA). The genotyping success rates were 98.7% (1917/1942) and 98.6% (1915/1942) for rs1744297 and rs2238732, respectively.

### In silico analysis of genetic variants

The exome sequencing data of 300 Japanese individuals from the Human Genetic Variation Database (HGVD) were used to identify candidates for genetic variants with a functional impact on PFAA concentrations [22, 23]. Pair-wise linkage disequilibrium (LD) coefficients (*r*^*2*^) were calculated using PLINK [20]. The impacts of the non-synonymous variants were predicted using the Ensembl Variant Effect Predictor [24], which is based on the SIFT [25] and PolyPhen [26] algorithms. Expression QTL (eQTL) analysis data (release version 8.0) were also downloaded from the HGVD [22].

## Results

### PFAA profiling

We measured the PFAA concentrations by an absolute quantification method using HPLC–ESI–MS [16–19]. The concentrations of 21 PFAAs were quantified successively in all of the samples (*N* = 2,094). The PFAA concentrations in the 1,338 samples used for GWAS are summarized with biochemical parameters in Table 1. The means, standard deviations, and ranges of the absolute concentrations were comparable to those obtained in an independent study in a Japanese population, except for arginine, glutamate, and ornithine [27]. The averaged levels of glutamate and ornithine higher, and that of arginine was lower, than in the previous study.

**Table 1:** Clinical characteristics and PFAA concentrations of the 1,338 subjects in this study

	*N* (%)	
Total	1338	
Women	873 (62.5%)	
Current smoker	256 (18.3%)	
Diabetes mellitus	63 (4.5%)	
Prevalent cardiovascular disease	49 (3.7%)	
Prevalent cancer	48 (3.4%)	
	**Mean (SD)**	**Range**
Age, years	49.8 (14.6)	30–75
Body-mass index, kg/cm2	22.1 (3.2)	14–41
Systolic blood pressure, mmHg	127.6 (17.4)	84–230
Diastolic blood pressure, mmHg	80.1 (11.1)	50–138
Blood glucose, mg/dL	92.1 (22.2)	68–572
HbA1c, %	5.38 (0.60)	4.23–14.22
Insulin, μIU/mL	6.15 (7.70)	0.77–118.00
Total cholesterol, mg/dL	203.0 (34.9)	86–338
HDL cholesterol, mg/dL	65.0 (16.6)	27–122
LDL cholesterol, mg/dL	120.4 (31.4)	26–240
Triglyceride, mg/dL	95.4 (68.0)	21–930
Free fatty acid, mEq/L	0.73 (0.29)	0.14–2.11
Total protein, g/dL	7.3 (0.4)	5.9–9.5
Albumin, g/dL	4.5 (0.2)	3.0–5.2
Amino acids, µM		
alanine (Ala)	319.3 (71.0)	169.5–646.4
alpha-amino-butyric acid (a-ABA)	16.4 (5.1)	4.2–59.9
arginine (Arg)	63.2 (18.2)	13.6–174.5
asparagine (Asn)	45.7 (8.0)	26.3–93.4
citrulline (Cit)	31.0 (8.2)	9.9–110.3
glutamate (Glu)	53.2 (19.3)	19.1–176.9
glutamine (Gln)	541.6 (69.2)	185.6–757.7
glycine (Gly)	225.6 (64.4)	90.7–717.9
histidine (His)	77.8 (10.1)	49.0–220.3
isoleucine (Ile)	56.5 (13.7)	24.0–119.9
leucine (Leu)	111.2 (23.0)	58.2–198.9
lysine (Lys)	171.5 (32.7)	84.7–326.4
methionine (Met)	21.9 (4.7)	11.5–60.3
ornithine (Orn)	82.8 (22.1)	31.3–181.8
phenylalanine (Phe)	54.5 (8.5)	34.3–95.9
proline (Pro)	133.5 (45.7)	53.7–559.9
serine (Ser)	112.7 (21.9)	42.0–215.2
threonine (Thr)	115.1 (28.1)	54.5–287.9
tryptophan (Trp)	50.1 (8.8)	27.2–81.9
tyrosine (Tyr)	56.8 (11.9)	27.1–130.9
valine (Val)	200.8 (41.0)	110.5–366.9

### QTL analysis of PFAAs (GWAS-1)

**Fig. 2:**
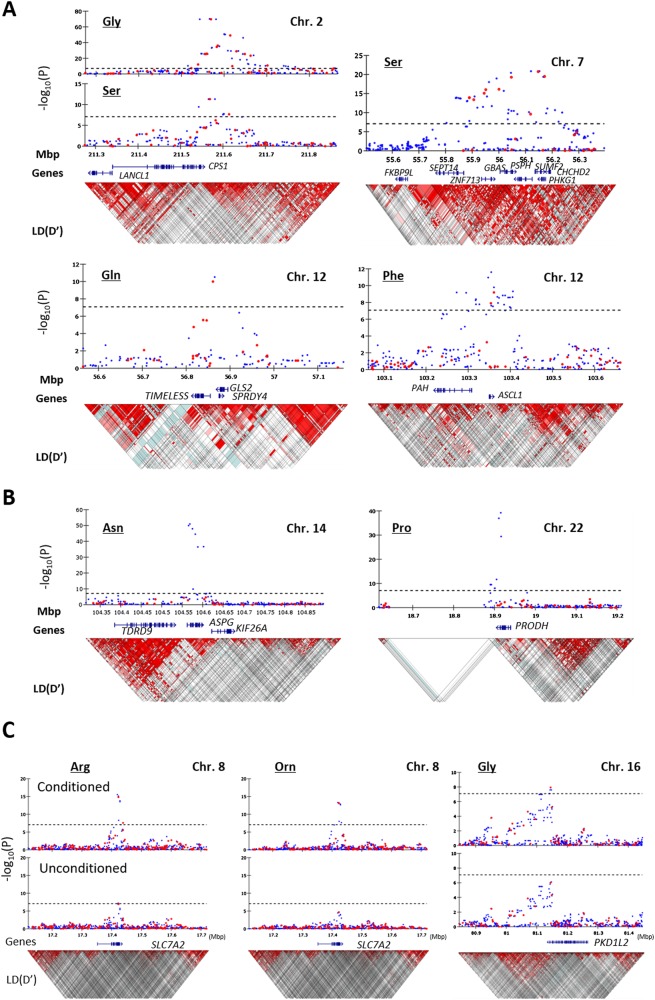
Regional association plots of the six loci significantly associated with PFAA concentrations (*n* = 1338). **a** Association was significantly identified from genotyped data. **b** Association was significantly identified after imputation. Chromosomal positions and *P* values for genotyped SNPs (red) and imputed SNPs (blue) are shown. **c** Chromosomal positions and *P* values for genotyped SNPs (red) and imputed SNPs (blue) of the conditional (upper) and unconditional (lower) analyses are shown. Dotted lines indicate the genome-wide significance threshold after Bonferroni correction. Brightness of the red color in the linkage disequilibrium (LD) blocks corresponds to the strength of LD

A flow diagram of the QC processes and QTL analyses is shown in Fig. 1. The first QTL analysis was conducted for each PFAA concentration, which was adjusted for sex and age after Box-Cox transformation, with 266,274 intersectional SNPs of 1338 samples (GWAS-1). Twenty-eight SNPs in four loci were significantly associated with glycine, serine, glutamine, and phenylalanine (Fig. 2a and Table 2). The strongest association was observed in the *CPS1* locus on chromosome 2 for the glycine concentration (rs12613336, *P* = 2.07 × 10^–70^). *CPS1* encodes mitochondrial carbamoyl-phosphate synthase 1 (CPS-I), a key enzyme in the urea cycle, which generates carbamoyl-phosphate from H_2_O, CO_2_, and ammonia. Two chromosomal loci showing significant associations with the serine concentration were identified: the *PSPH* locus on chromosome 7 (rs13244654, *P* = 1.80 ×10^–21^) and the *CPS1* locus, which was also associated with the glycine concentration (rs12613336, *P* = 4.77 × 10^–12^). *PSPH* encodes phosphoserine phosphatase (PSPH), which catalyzes the hydrolysis of 3-phosphoserine to generate serine. In addition, the *GLS2* locus and the *PAH* locus on chromosome 12 were associated with the concentration of glutamine (rs7302925, *P* = 9.73 × 10^–11^), and phenylalanine (rs17450273, *P* = 6.60 × 10^–10^), respectively. *GLS2* encodes glutaminase, which catalyzes the hydrolysis of glutamine to glutamate and ammonia, and *PAH* encodes phenylalanine hydroxylase.

**Table 2:** Genetic variants associated with PFAAs in Japanese

Trait	Locus	SNP ID	Chr.^a^	Position	Beta (SE^b^)	*r* ^2^	*P* value	Ref. (A1) / Var. (A2).	Freq. (A1)	Annotation
GWAS-1										
Gly	*CPS1*	rs12613336	2	211,569,399	0.90 (0.05)	0.21	2.07 × 10^–70^	T/C	0.90	Intergenic
Ser	*CPS1*	rs12613336	2	211,569,399	0.34 (0.05)	0.04	4.77 × 10^–12^	T/C	0.90	Intergenic
Ser	*PSPH*	rs13244654	7	56,146,956	−0.36 (0.04)	0.07	1.80 × 10^–21^	T/C	0.90	Intronic
Gln	*GLS2*	rs7302925	12	56,861,458	−0.39 (0.06)	0.03	9.73 × 10^–11^	A/G	0.85	Upstream
Phe	*PAH*	rs17450273	12	103,361,379	0.35 (0.06)	0.03	6.60 × 10^–10^	C/A	0.69	Intergenic
Asn	*ASPG* ^c^	rs1744297	14	104,568,472	0.82 (0.05)	0.16	1.30 × 10^–51^	T/C	0.87	Intronic
Pro	*PRODH* ^c^	rs2238732	22	18,915,347	0.69 (0.05)	0.12	5.96 × 10^–40^	C/T	0.87	Intronic
GWAS-2										
Arg	*SLC7A2*	rs56335308	8	17,419,461	−0.43 (0.05)	0.05	2.64 × 10^–16^	G/A	0.93	Exonic, Non-synonymous
Orn	*SLC7A2*	rs56335308	8	17,419,461	−0.35 (0.05)	0.04	4.70 × 10^–14^	G/A	0.93	Exonic, Non-synonymous
Gly	*PKD1L2*	rs8059153	16	81,145,675	−0.21 (0.04)	0.02	1.46 × 10^–8^	T/C	0.93	Intronic

^a^chromosome

^b^standard error

^c^imputation was performed using the genotyping results of 665 samples that were unrelated to those used for the present study

Next, we performed imputation using the genotyping results of 665 samples that were unrelated to those used for the present GWA study. The additional imputed genotypes of 1,021,928 SNPs were used for the QTL analysis. We identified two additional chromosomal loci in which multiple SNP markers showed a significant association with PFAA concentrations (Fig. 2b and Table 2): the *ASPG* (putative asparaginase) locus on chromosome 14 for asparagine (rs1744297, *P* = 1.30 × 10^–51^) and the *PRODH* (proline dehydrogenase) locus on chromosome 22 for proline (rs2238732, *P* = 5.96 × 10^–40^). To confirm these associations, these two SNPs were genotyped for the same DNA samples using the Taqman assay (Table 3). We obtained *P* = 2.36 × 10^–48^ for rs1744297 and *P* = 5.78 × 10^–36^ for rs2238732, and the concordance rates between the imputed and directly genotyped SNPs were 98.6% (1300/1319) and 97.6% (1289/1321) for rs1744297 and rs2238732, respectively.

The imputation analysis identified six additional chromosomal loci with potential associations. However, only one SNP marker showed a significant association for each locus, so no further analysis was performed. The 151 SNPs showing significant associations either by genotyping or by imputation are listed in Table S1.

### QTL analysis of PFAAs conditioned on the other amino acids (GWAS-2)

We next conducted QTL analysis using the concentration of each PFAA adjusted using the other 20 PFAAs as covariates (GWAS-2 in Fig. 1). The optimal regression models for each PFAA were constructed using a step-wise variable selection method (Table S2). Two additional association loci were identified by the conditional QTL analysis (Fig. 2c and Table 2). The strongest association was observed at a non-synonymous variant, rs56335308, in the *SLC7A2* (a solute carrier family 7, cationic amino acid transporter) gene on chromosome 8 for arginine (*P* = 2.64 × 10^–16^) and ornithine (*P* = 4.70 × 10^–14^). The other was the *PKD1L2* locus on chromosome 16 for glycine (rs8059153, *P* = 1.46 × 10^–8^). This gene encodes a member of the polycystin protein family. In addition, an association was found between rs2238732 in the *PRODH* locus and the alanine concentration (*P* = 7.10 × 10^–11^). On the other hand, the significant association between serine and the *CPS1* locus disappeared (*P* *=* 0.63). The other six associations maintained significant levels after the conditional analysis. The 209 SNPs identified as significant in GWAS-2 are listed in Table S3.

### QTL analysis of PFAAs adjusted conditioned on one of the other amino acids (GWAS-3)

**Fig. 3:**
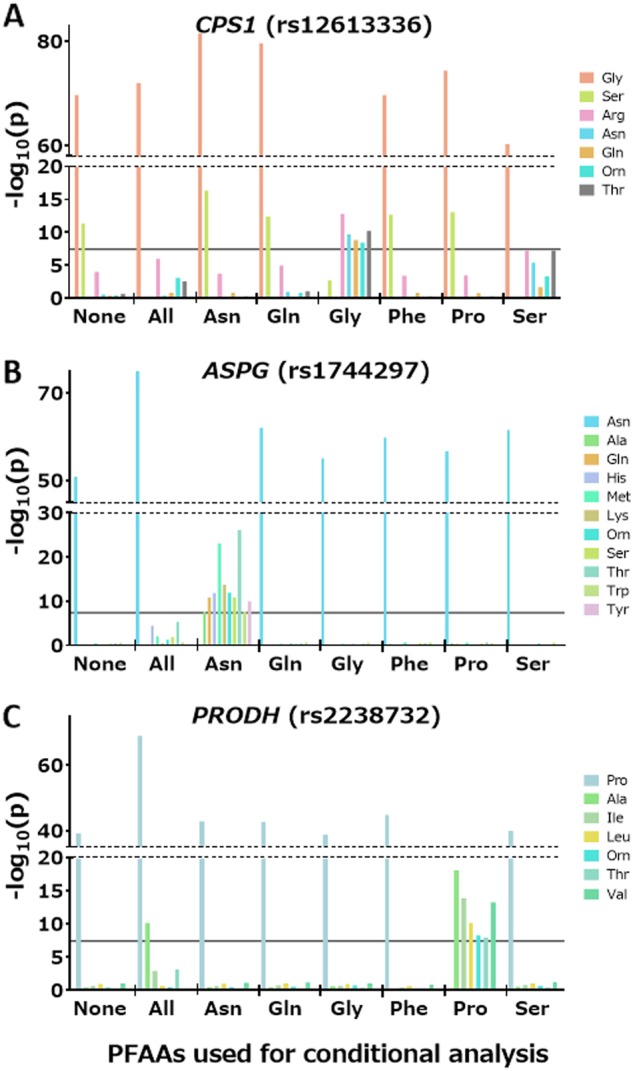
Strength of the associations of three loci (*CPS1*, *ASPG*, and *PRODH*) conditioned on other PFAA concentrations. *P* values for the SNPs conditioned on other PFAA concentrations are plotted as bars

We also conducted QTL analyses of 21 PFAAs conditioned on one of the six PFAAs (asparagine, glutamine, glycine, phenylalanine, proline, and serine) identified as significant in the above studies (GWAS-3 in Fig. 1). The significant association between serine and the *CPS1* locus disappeared when glycine was used for the adjustment (*P* *=* 0.002) (Fig. 3a). In contrast, significant associations between five PFAAs (arginine, asparagine, glutamine, ornithine, and threonine) and the *CPS1* locus were still apparent when glycine was used as a covariate (Fig. 3a).

When asparagine was used as a covariate, rs1744297 in the *ASPG* locus showed additional associations with ten PFAAs: alanine, glutamine, histidine, lysine, methionine, ornithine, serine, threonine, tryptophan, and tyrosine (Fig. 3b). Rs2238732 in the *PRODH* locus also showed an association with seven PFAAs (alanine, isoleucine, leucine, ornithine, threonine, tyrosine, and valine) (Fig. 3c) using proline as a covariate. Similarly, significant associations were observed in *PSPH* for threonine using serine (*P* = 3.82 × 10^–8^) and in *PAH* for methionine using phenylalanine (*P* = 1.91 × 10^–9^) as a covariate. All of the statistics in GWAS-3 are listed in Table S4.

### In silico analysis for the functional interpretation of the association between identified SNPs and PFAA concentrations

In the above analyses, three of the eight loci that showed significant associations, namely, rs56335308 in *GLS2*, rs2657879 in *PRODH*, and rs450046 in *SLC7A2*, were non-synonymous variations with potential functional effects on PFAA concentrations (Table S1). We tried to identify non-synonymous SNPs that were in strong LD with the SNPs showing significant associations within the eight loci in the above analysis, by conducting an in silico analysis using pair-wise LD information in the HGVD exome sequencing data set [23]. Among the 223 SNPs with LD coefficients (*r*^2^) greater than 0.8 with SNPs significantly associated with PFAAs, four non-synonymous variants were identified: rs1047891 in *CPS1*, rs8012505 in *ASPG*, rs8054182 in *PKD1L2*, and rs5747933 in *PRODH* (Table S5). The predicted functional impacts of these variants were not deleterious to the gene products except for *ASPG*. There were no non-synonymous variants for the *PSPH* or *PAH* gene.

We next investigated whether these SNPs within the eight loci identified in the current study or those that were in strong LD with them influenced gene expression using the HGVD expression QTL (eQTL) information [22]. We found that rs4948073, which was located approximately 240-kb downstream of the *PSPH* gene, showed the strongest association with the expression level of the *PSPH* gene (*P* = 3.03 × 10^–46^). There were no significant eQTLs for the other six loci.

## Discussion

**Fig. 4:**
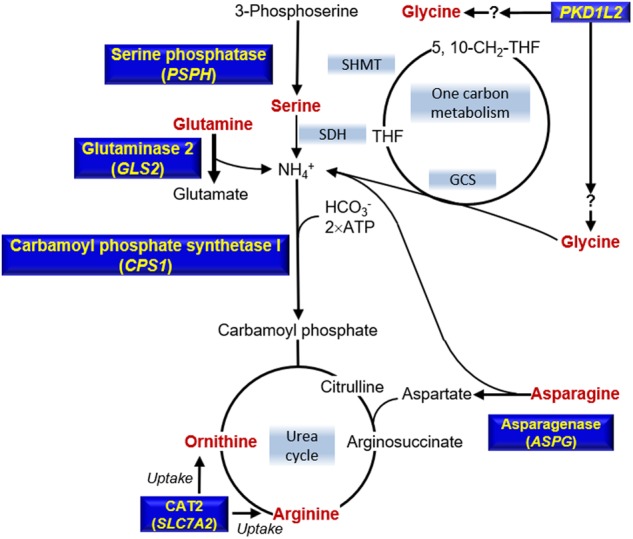
Metabolic pathways relevant to genotype-PFAA associations. The six PFAAs (red) were associated with genotypes in the genes (yellow). *THF* tetrahydrofuran, *5-CH*_*3*_*-THF* 5-methyltetrahydrofolate, *5,10-CH*_*2*_*-THF* 5,10-methylenetetrahydrofuran, *NH*_*4*_^*+*^ ammonium ion, *GCS* glycine cleavage system, *SDH* serine dehydratase, *SHMT* serine hydroxymethyltransferase

In this study, we conducted a QTL analysis of the absolute concentrations of PFAAs quantified by LC-MS technology. The concentration of a PFAA can be influenced by other PFAAs within the same metabolic pathway. Therefore, it is also important to perform conditional QTL analysis considering the amino acids’ metabolic pathways when identifying the genetic determinants of PFAA concentrations. Notably, here we identified two additional genetic loci associated with the concentrations of arginine, ornithine, and glycine (Table S2). One of the identified genes, *SLC7A2* was associated with arginine and ornithine. This protein is known to transport plasma arginine into cells for protein synthesis and to convert arginine into ornithine or nitric oxide [28]. SLC7A family members, such as SLC7A5, SLC7A6, and SLC7A9, are associated with plasma tryptophan, lysine, and arginine, respectively [9, 13, 29, 30]. Thus, it is likely that genetic variations of *SLC7A2* would affect the plasma concentration of arginine and ornithine. The other identified gene, *PKD1L2*, which showed an association with glycine concentration, encodes polycystic kidney disease protein 1-like 2. Previous studies suggested that genetic variation of the *PKD1L2* gene may be associated with high-density lipoprotein cholesterol [31, 32]. Rs8054182, which has strong LD with rs8059153 (*r*^2^ = 0.989), introduces an amino acid change from methionine to isoleucine at position 1630, which is in the conserved ion channel pore region [33]. It is therefore possible to speculate that PKD1L2 acts as part of a glycine transporter (Fig. 4).

Significant associations of *CPS1* with the plasma levels of arginine, asparagine, glutamine, ornithine, and threonine were observed only after being conditioned on glycine (Fig. 3a). Asparagine and glutamine syntheses have CPS-I in their upstream pathway (Fig. 4). Threonine is also involved in the ammonia-generating reaction mediated by L-serine dehydratase/L-threonine deaminase [34]. Both arginine and ornithine are involved in the urea cycle, which is the downstream pathway of CPS-I (Fig. 4). These mechanisms suggest that the plasma concentrations of these five PFAAs are influenced by the enzymatic activity of CPS-I. Similarly, associations exist for *ASPG* for ten PFAAs were obtained only after being conditioned on asparagine (Fig. 3b). Rs8012505, a non-synonymous SNP that has strong LD with rs1744297, is located at the provisional cytoplasmic asparaginase I (ansA) domain and changes serine to arginine at position 344. We speculate that ASPG can use these ten PFAAs as substrates for deamination. Similarly, significant associations of *PRODH* for seven PFAAs were obtained only after being conditioned on proline, suggesting that PRODH can use them as substrates (Fig. 3c). In some situations, use of heritable covariates might introduce unintended bias into estimate [35]. Direct enzymatic verification whether ASPG and PRODH can catalyze other amino acids than asparagine and proline, respectively, will be desirable to confirm our speculations.

**Table 3:** Direct genotyping assay of *ASPG* and *PRODH* loci

Locus (rs ID)	Genotyped samples				Validation samples				Combined data set			
	Beta(SE^a^)	*r* ^2^	*P* value	*N*	Beta(SE^a^)	*r* ^2^	*P* value	*N*	Beta(SE^a^)	*r* ^2^	*P* value	*N*
*ASPG* (rs1744297)	0.79 (0.05)	0.15	2.36 × 10^–48^	1319	0.83 (0.07)	0.19	2.40 × 10^–28^	598	0.81 (0.04)	0.16	1.04 × 10^–74^	1917
*PRODH* (rs2238732)	0.64 (0.05)	0.11	5.78 × 10^–36^	1321	0.67 (0.07)	0.14	1.21 × 10^–20^	594	0.65 (0.04)	0.12	2.33 × 10^–54^	1915

^a^standard error

We also demonstrated that the conditional QTL analysis is useful for determining the metabolic pathway predominantly used for PFAA metabolism. For example, the concentration of plasma glycine is correlated with that of serine (*r* = 0.54), and the significant association between serine and *CPS1* in the unconditional QTL analysis disappeared when conditioned on glycine (Fig. 3a). In contrast, the significant association between glycine and *CPS1* was not affected by the analysis conditioned on serine (Fig. 3a). CPS-I is considered an entrance to the urea cycle, which detoxifies the ammonia that is produced by amino acid degradation. Two separate pathways that generate ammonia are likely to be involved in this process. The first is the conversion of glycine to ammonia catalyzed by the glycine cleavage system (GCS) with tetrahydrofolate production [36]. The second is the conversion of serine to ammonia and pyruvate, which is catalyzed by serine dehydratase [37]. In addition, glycine and serine are reversibly converted to each other via serine hydroxymethyltransferase [38]. The study of hyperglycinemia, an inborn deficiency of GCS, revealed that GCS plays a critical role in both glycine and serine catabolism in the liver [36]. The results of the conditional QTL analysis in the present study were consistent with previous clinical observations.

It is still unknown whether common variants that influence the concentrations of plasma amino acids are associated with risks of lifestyle-related metabolic diseases. For example, although the plasma glycine concentration is associated with an increased risk of diabetes [7, 8, 39], no genetic variants that are significantly associated with a risk of diabetes have been identified within the *CPS1* locus [12]. Further longitudinal studies with increased sample sizes are needed to assess whether the PFAA concentrations can be used as intermediate biomarkers for metabolic disease risk under a variety of genetic backgrounds.

## Electronic supplementary material

Supplementary table S1

Supplementary table S2

Supplementary table S3

Supplementary table S4

Supplementary table S5
