# Supplementary material for: Genetic basis for plasma amino acid concentrations based on absolute quantification: a genome-wide association study in the Japanese population
Source: Eur J Hum Genet. 2019 Jan 18;27(4):621–30. doi: 10.1038/s41431-018-0296-y (PMC6460579; doi:10.1038/s41431-018-0296-y)
Supplement: Supplementary file 1 — Supplementary table S1 [file 41431_2018_296_MOESM1_ESM.docx]

Supplementary table S1. All the significant associations identified in GWAS-1.

| **Trait** | **Locus** | **SNP*^a^* 　　(rs ID)** | **Chr*^b^*** | **Position** | **Beta (SE*^c^*)** | ***r*^2^** | ***P* value** | **Ref. (A1) /Var. (A2).** | **Freq. (A1)** | **Annotation** |
| --- | --- | --- | --- | --- | --- | --- | --- | --- | --- | --- |
| Met | CCT4 | rs62149891 | 2 | 62105134 | -0.23(0.04) | 0.03 | 5.55e-09 | A/G | 0.29 | Intronic |
| Thr | *CCT4* | rs62149891 | 2 | 62105134 | -0.23(0.04) | 0.02 | 2.07e-08 | A/G | 0.29 | Intronic |
| Gly | *CPS1* | rs34449727 | 2 | 211514995 | 0.44(0.07) | 0.03 | 4.54e-11 | G/T | 0.10 | Intronic |
| **Gly** | ***CPS1*** | **rs4567871** | **2** | **211539302** | **0.30(0.04)** | **0.04** | **1.47E-12** | **C/T** | 0.30 | **Intronic** |
| Gly | *CPS1* | rs715 | 2 | 211543055 | 0.88(0.05) | 0.21 | 2.49e-70 | T/C | 0.17 | 3'UTR |
| Ser | *CPS1* | rs715 | 2 | 211543055 | 0.31(0.05) | 0.03 | 3.46e-10 | T/C | 0.17 | 3'UTR |
| Gly | *CPS1* | rs4142152 | 2 | 211546043 | 0.42(0.04) | 0.08 | 1.65e-26 | C/T | 0.35 | Intergenic |
| Gly | *CPS1* | rs13386028 | 2 | 211547400 | 0.42(0.04) | 0.08 | 1.65e-26 | C/A | 0.35 | Intergenic |
| Gly | *CPS1* | rs4672588 | 2 | 211553159 | 0.41(0.04) | 0.08 | 5.16e-26 | T/C | 0.35 | Intergenic |
| **Gly** | ***CPS1*** | **rs2887915** | **2** | **211554574** | **0.41(0.04)** | **0.08** | **5.16E-26** | **C/T** | 0.34 | **Intergenic** |
| **Gly** | ***CPS1*** | **rs10490320** | **2** | **211556975** | **0.42(0.04)** | **0.08** | **3.24E-26** | **T/C** | 0.34 | **Intergenic** |
| **Gly** | ***CPS1*** | **rs2371023** | **2** | **211559526** | **0.42(0.04)** | **0.08** | **2.00E-26** | **G/A** | 0.34 | **Intergenic** |
| Gly | *CPS1* | rs79832648 | 2 | 211561489 | 0.42(0.04) | 0.08 | 3.55e-26 | C/T | 0.35 | Intergenic |
| Gly | *CPS1* | rs4673546 | 2 | 211565692 | 0.90(0.05) | 0.21 | 6.32e-71 | C/T | 0.16 | Intergenic |
| Ser | *CPS1* | rs4673546 | 2 | 211565692 | 0.34(0.05) | 0.03 | 5.50e-12 | C/T | 0.16 | Intergenic |
| **Gly** | ***CPS1*** | **rs12613336** | **2** | **211569399** | **0.90(0.05)** | **0.21** | **2.07E-70** | **T/C** | 0.15 | **Intergenic** |
| **Ser** | ***CPS1*** | **rs12613336** | **2** | **211569399** | **0.34(0.05)** | **0.04** | **4.70E-12** | **T/C** | 0.15 | **Intergenic** |
| Gly | *CPS1* | rs1016396 | 2 | 211570448 | 0.50(0.04) | 0.10 | 1.00e-33 | C/T | 0.28 | Intergenic |
| Gly | *CPS1* | rs4673547 | 2 | 211571520 | 0.50(0.04) | 0.11 | 1.99e-34 | A/G | 0.29 | Intergenic |
| Gly | *CPS1* | rs3815630 | 2 | 211581142 | 0.90(0.05) | 0.21 | 2.07e-70 | T/C | 0.15 | Intergenic |
| Ser | *CPS1* | rs3815630 | 2 | 211581142 | 0.34(0.05) | 0.04 | 4.77e-12 | T/C | 0.15 | Intergenic |
| **Gly** | ***CPS1*** | **rs7583500** | **2** | **211583066** | **0.52(0.04)** | **0.11** | **3.02E-35** | **T/C** | 0.27 | **Intergenic** |
| **Gly** | ***CPS1*** | **rs1861897** | **2** | **211585271** | **0.53(0.04)** | **0.11** | **6.21E-37** | **C/T** | 0.27 | **Intergenic** |
| Gly | *CPS1* | rs12464097 | 2 | 211589248 | 0.52(0.04) | 0.11 | 5.57e-36 | A/C | 0.28 | Intergenic |
| Gly | *CPS1* | rs10932355 | 2 | 211600311 | 0.82(0.05) | 0.16 | 1.87e-51 | A/G | 0.14 | Intergenic |
| Ser | *CPS1* | rs10932355 | 2 | 211600311 | 0.29(0.05) | 0.02 | 2.45e-08 | A/G | 0.14 | Intergenic |
| Gly | *CPS1* | rs2160847 | 2 | 211601116 | 0.82(0.05) | 0.16 | 6.33e-51 | T/C | 0.14 | Intergenic |
| Ser | *CPS1* | rs2160847 | 2 | 211601116 | 0.30(0.05) | 0.02 | 1.91e-08 | T/C | 0.14 | Intergenic |
| Gly | *CPS1* | rs16844874 | 2 | 211602416 | 0.82(0.05) | 0.16 | 6.33e-51 | T/C | 0.14 | Intergenic |
| Ser | *CPS1* | rs16844874 | 2 | 211602416 | 0.30(0.05) | 0.02 | 1.91e-08 | T/C | 0.14 | Intergenic |
| Gly | *CPS1* | rs10515953 | 2 | 211608583 | 0.46(0.04) | 0.09 | 2.35e-30 | G/A | 0.31 | Intergenic |
| **Gly** | ***CPS1*** | **rs10206976** | **2** | **211614638** | **0.82(0.05)** | **0.15** | **7.35E-50** | **G/T** | 0.13 | **Intergenic** |
| **Ser** | ***CPS1*** | **rs10206976** | **2** | **211614638** | **0.30(0.05)** | **0.02** | **2.17E-08** | **G/T** | 0.13 | **Intergenic** |
| Gly | *CPS1* | rs4673554 | 2 | 211616067 | 0.46(0.04) | 0.09 | 6.13e-30 | G/T | 0.31 | Intergenic |
| Gly | *CPS1* | rs2287411 | 2 | 211618187 | 0.40(0.04) | 0.06 | 1.16e-20 | C/G | 0.28 | Intergenic |
| **Gly** | ***CPS1*** | **rs11689207** | **2** | **211619571** | **0.71(0.11)** | **0.03** | **9.36E-11** | **A/G** | 0.03 | **Intergenic** |
| Gly | *CPS1* | rs10490327 | 2 | 211619881 | 0.71(0.11) | 0.03 | 9.11e-11 | G/A | 0.04 | Intergenic |
| **Gly** | ***CPS1*** | **rs2371074** | **2** | **211628462** | **0.46(0.04)** | **0.09** | **1.41E-29** | **C/T** | 0.31 | **Intergenic** |
| Gly | *CPS1* | rs6736693 | 2 | 211629715 | 0.46(0.04) | 0.09 | 1.07e-29 | A/G | 0.31 | Intergenic |
| Gly | *CPS1* | rs10208743 | 2 | 211633505 | 0.46(0.04) | 0.09 | 3.59e-30 | A/G | 0.31 | Intergenic |
| Gly | *CPS1* | rs10201195 | 2 | 211637823 | 0.38(0.04) | 0.06 | 6.10e-19 | C/A | 0.27 | Intergenic |
| Gly | *CPS1* | rs969816 | 2 | 211643761 | 0.42(0.04) | 0.08 | 3.20e-26 | A/G | 0.34 | Intergenic |
| Gly | *CPS1* | rs10172053 | 2 | 211644647 | 0.79(0.05) | 0.14 | 1.44e-46 | T/G | 0.14 | Intergenic |
| Gly | *CPS1* | rs10932361 | 2 | 211647733 | 0.42(0.04) | 0.08 | 5.11e-26 | T/C | 0.34 | Intergenic |
| **Gly** | ***CPS1*** | **rs4672596** | **2** | **211648806** | **0.42(0.04)** | **0.08** | **5.29E-26** | **T/C** | 0.33 | **Intergenic** |
| Gly | *CPS1* | rs7568719 | 2 | 211653368 | 0.38(0.04) | 0.06 | 9.44e-19 | C/T | 0.28 | Intergenic |
| Gly | *CPS1* | rs6758081 | 2 | 211655965 | 0.34(0.04) | 0.04 | 8.26e-14 | A/G | 0.25 | Intergenic |
| Gly | *CPS1* | rs10205894 | 2 | 211657098 | 0.33(0.04) | 0.04 | 4.22e-13 | A/G | 0.24 | Intergenic |
| **Gly** | ***CPS1*** | **rs4673558** | **2** | **211661897** | **0.52(0.05)** | **0.07** | **9.40E-24** | **C/T** | 0.16 | **Intergenic** |
| Gly | *CPS1* | rs12998006 | 2 | 211669006 | 0.53(0.05) | 0.07 | 2.03e-24 | T/C | 0.16 | Intergenic |
| Gly | *CPS1* | rs16824978 | 2 | 211672061 | 0.32(0.04) | 0.04 | 1.53e-12 | C/T | 0.25 | Intergenic |
| Gly | *CPS1* | rs13012874 | 2 | 211673049 | 0.32(0.04) | 0.04 | 3.46e-12 | G/A | 0.24 | Intergenic |
| Gly | *CPS1* | rs36037305 | 2 | 211684149 | 0.35(0.05) | 0.03 | 4.99e-11 | A/G | 0.15 | Intergenic |
| Gly | *CPS1* | rs62202017 | 2 | 211697251 | 0.35(0.05) | 0.03 | 3.49e-11 | T/C | 0.15 | Intergenic |
| **Gly** | ***CPS1*** | **rs13019409** | **2** | **211701811** | **0.35(0.05)** | **0.03** | **1.89E-11** | **A/G** | 0.15 | **Intergenic** |
| Gly | *CPS1* | rs6726552 | 2 | 211740016 | 0.32(0.05) | 0.03 | 3.13e-11 | G/A | 0.20 | Intergenic |
| Gly | *CPS1* | rs6759308 | 2 | 211744633 | 0.31(0.05) | 0.03 | 1.06e-10 | G/T | 0.20 | Intergenic |
| Gly | *CPS1* | rs4310999 | 2 | 211750298 | 0.34(0.05) | 0.03 | 3.92e-10 | G/A | 0.14 | Intergenic |
| Gly | *CPS1* | rs4338916 | 2 | 211766532 | 0.34(0.05) | 0.03 | 5.70e-10 | G/T | 0.14 | Intergenic |
| Gly | *RPS27P10* | rs1657863 | 2 | 211859726 | 0.26(0.05) | 0.02 | 3.23e-08 | T/G | 0.21 | Intergenic |
| Leu | *RPL23AP42* | rs117936590 | 3 | 161138859 | 0.52(0.09) | 0.02 | 2.07e-08 | C/T | 0.04 | Intergenic |
| His | *PRKAR1B* | rs75444260 | 7 | 675581 | 0.42(0.07) | 0.02 | 6.59E-09 | A/G | 0.11 | Intronic |
| Trp | *PRKAR1B* | rs75444260 | 7 | 675581 | 0.39(0.07) | 0.02 | 2.75E-08 | A/G | 0.11 | Intronic |
| **Ser** | ***PSPHL*** | **rs11766051** | **7** | **55838545** | **-0.28(0.04)** | **0.04** | **8.70E-15** | **G/T** | 0.41 | **NA** |
| Ser | *PSPHL* | rs11976304 | 7 | 55840503 | -0.27(0.04) | 0.04 | 1.26e-14 | T/C | 0.41 | NA |
| Ser | *PSPHL* | rs6700 | 7 | 55841188 | -0.27(0.04) | 0.04 | 1.26e-14 | C/T | 0.41 | NA |
| Ser | *PSPHL* | rs6966061 | 7 | 55848790 | -0.27(0.04) | 0.04 | 1.46e-14 | A/G | 0.41 | Intergenic |
| Ser | *SEPT14* | rs11982736 | 7 | 55855180 | -0.27(0.03) | 0.04 | 1.78e-14 | G/A | 0.44 | Intergenic |
| Ser | *SEPT14* | rs9642404 | 7 | 55856799 | -0.27(0.04) | 0.04 | 1.94e-14 | G/A | 0.41 | Intergenic |
| Ser | *SEPT14* | rs28803557 | 7 | 55880211 | -0.26(0.04) | 0.04 | 9.71e-14 | G/A | 0.42 | Intronic |
| Ser | *SEPT14* | rs10233558 | 7 | 55881666 | -0.27(0.04) | 0.04 | 8.40e-14 | C/T | 0.42 | Intronic |
| **Ser** | ***SEPT14*** | **rs4588807** | **7** | **55889109** | **-0.27(0.03)** | **0.04** | **1.33E-14** | **A/G** | 0.47 | **Intronic** |
| **Ser** | ***SEPT14*** | **rs1113765** | **7** | **55889334** | **-0.27(0.03)** | **0.04** | **1.14E-14** | **G/A** | 0.47 | **Intronic** |
| Ser | *SEPT14* | rs76308007 | 7 | 55889433 | 0.25(0.04) | 0.02 | 1.68e-08 | A/G | 0.21 | Intronic |
| **Ser** | ***SEPT14*** | **rs10230845** | **7** | **55905896** | **-0.27(0.04)** | **0.04** | **4.26E-14** | **C/T** | 0.41 | **Intronic** |
| Ser | *SEPT14* | rs10239528 | 7 | 55908211 | -0.27(0.03) | 0.04 | 6.35e-15 | C/A | 0.46 | Intronic |
| Ser | *SEPT14* | rs4476962 | 7 | 55919938 | 0.26(0.04) | 0.03 | 5.41e-12 | C/T | 0.34 | Intronic |
| Ser | *SEPT14* | rs13233754 | 7 | 55941077 | 0.28(0.04) | 0.04 | 3.28e-12 | G/A | 0.26 | Intergenic |
| **Ser** | ***SEPT14*** | **rs6593287** | **7** | **55944185** | **-0.28(0.03)** | **0.05** | **7.69E-16** | **G/A** | 0.46 | **Intergenic** |
| **Ser** | ***SEPT14*** | **rs11761352** | **7** | **55950947** | **-0.30(0.04)** | **0.05** | **9.32E-17** | **C/A** | 0.38 | **Intergenic** |
| Ser | *SEPT14* | rs6947698 | 7 | 55954993 | -0.27(0.03) | 0.04 | 6.07e-15 | G/C | 0.48 | Intergenic |
| Ser | *ZNF713* | rs10271662 | 7 | 55962907 | -0.32(0.04) | 0.06 | 9.27e-19 | A/C | 0.36 | Intergenic |
| Ser | *ZNF713* | rs35304280 | 7 | 55972857 | 0.30(0.04) | 0.04 | 2.05e-12 | A/C | 0.22 | Intergenic |
| Ser | *ZNF713* | rs2135116 | 7 | 55991144 | -0.33(0.04) | 0.06 | 9.75e-20 | A/G | 0.36 | Intronic |
| **Ser** | ***ZNF713*** | **rs13222366** | **7** | **55999702** | **-0.29(0.03)** | **0.05** | **7.17E-17** | **G/A** | 0.48 | **Intronic** |
| Ser | *MRPS17* | rs12671091 | 7 | 56018666 | -0.29(0.03) | 0.05 | 1.78e-16 | T/C | 0.48 | Upstream |
| Ser | *GBAS* | rs11238386 | 7 | 56033141 | 0.22(0.04) | 0.03 | 5.89e-09 | A/C | 0.33 | Intronic |
| Ser | *GBAS* | rs34929372 | 7 | 56034559 | 0.24(0.04) | 0.03 | 1.67e-10 | T/C | 0.33 | Intronic |
| Ser | *GBAS* | rs4360246 | 7 | 56036606 | 0.28(0.04) | 0.03 | 1.70e-11 | G/C | 0.24 | Intronic |
| Ser | *GBAS* | rs4535700 | 7 | 56045448 | -0.34(0.04) | 0.06 | 3.75e-21 | T/C | 0.37 | Intronic |
| Ser | *GBAS* | rs73343719 | 7 | 56046421 | 0.27(0.04) | 0.03 | 3.39e-11 | A/G | 0.24 | Intronic |
| **Ser** | ***GBAS*** | **rs4543497** | **7** | **56047215** | **-0.34(0.04)** | **0.06** | **4.72E-20** | **T/C** | 0.36 | **Intronic** |
| Ser | *GBAS* | rs13239795 | 7 | 56055327 | 0.29(0.04) | 0.03 | 3.22e-11 | C/T | 0.21 | Intronic |
| Ser | *GBAS* | rs13245637 | 7 | 56068980 | 0.24(0.04) | 0.03 | 2.27e-10 | C/T | 0.34 | Intergenic |
| Ser | *PSPH* | rs35210477 | 7 | 56112217 | 0.29(0.04) | 0.03 | 6.78e-11 | A/G | 0.20 | Intronic |
| **Ser** | ***PSPH*** | **rs6593296** | **7** | **56118293** | **0.26(0.04)** | **0.03** | **2.23E-10** | **C/T** | 0.24 | **Intronic** |
| Ser | *CCT6A* | rs7793921 | 7 | 56120881 | -0.34(0.03) | 0.07 | 1.59e-21 | G/A | 0.39 | Intronic |
| Ser | *SUMF2* | rs13238899 | 7 | 56140618 | 0.22(0.04) | 0.02 | 2.90e-08 | T/G | 0.26 | Intronic |
| **Ser** | ***SUMF2*** | **rs13244654** | **7** | **56146956** | **-0.36(0.04)** | **0.07** | **1.80E-21** | **T/C** | 0.31 | **Intronic** |
| Ser | *PHKG1* | rs2242508 | 7 | 56151489 | -0.36(0.04) | 0.07 | 1.55e-21 | A/G | 0.33 | Intronic |
| Ser | *CHCHD2* | rs4948106 | 7 | 56167595 | -0.33(0.03) | 0.06 | 4.29e-20 | T/G | 0.38 | Intergenic |
| **Ser** | ***CHCHD2*** | **rs816411** | **7** | **56171489** | **-0.33(0.03)** | **0.06** | **3.69E-20** | **C/T** | 0.37 | **Intronic** |
| Ser | *CHCHD2* | rs35557048 | 7 | 56176832 | 0.28(0.04) | 0.03 | 1.65e-10 | G/A | 0.21 | Intergenic |
| Ser | *LOC389493* | rs7782243 | 7 | 56181845 | -0.27(0.04) | 0.03 | 7.96e-12 | A/G | 0.28 | Downstream |
| Ser | *LOC442309* | rs2538054 | 7 | 56210441 | -0.29(0.04) | 0.03 | 1.95e-11 | A/G | 0.22 | Intergenic |
| Ser | *LOC442309* | rs34148685 | 7 | 56223739 | 0.25(0.04) | 0.02 | 2.42e-08 | T/C | 0.20 | Intergenic |
| Ser | *LOC100130909* | rs816396 | 7 | 56237280 | -0.29(0.04) | 0.03 | 8.32e-11 | T/C | 0.20 | Intergenic |
| **Gln** | ***SPRYD4*** | **rs7302925** | **12** | **56861458** | **-0.39(0.06)** | **0.03** | **9.73E-11** | **A/G** | 0.10 | **Upstream** |
| Gln | *GLS2* | rs2657879 | 12 | 56865338 | -0.41(0.06) | 0.03 | 2.96e-11 | A/G | 0.09 | Exonic,Non-synonymous |
| Leu | *NAV3* | rs10437868 | 12 | 77902318 | 0.79(0.14) | 0.02 | 2.59e-08 | T/C | 0.02 | Intergenic |
| Phe | *PAH* | rs1718302 | 12 | 103272686 | 0.31(0.05) | 0.03 | 6.24e-10 | G/A | 0.14 | Intronic |
| Phe | *PAH* | rs78985461 | 12 | 103295636 | 0.32(0.05) | 0.03 | 6.98e-10 | G/A | 0.12 | Intronic |
| Phe | *PAH* | rs34281125 | 12 | 103317416 | 0.30(0.05) | 0.03 | 5.52e-09 | C/T | 0.13 | Intergenic |
| Phe | *PAH* | rs71466246 | 12 | 103318463 | 0.30(0.05) | 0.03 | 4.21e-09 | G/A | 0.13 | Intergenic |
| Phe | *PAH* | rs34732688 | 12 | 103318823 | 0.28(0.05) | 0.02 | 1.71e-08 | A/G | 0.14 | Intergenic |
| Phe | *PAH* | rs17547025 | 12 | 103326270 | 0.28(0.05) | 0.02 | 1.71e-08 | A/C | 0.14 | Intergenic |
| Phe | *ASCL1* | rs7973625 | 12 | 103347511 | 0.41(0.06) | 0.03 | 1.11e-11 | T/C | 0.09 | Intergenic |
| **Phe** | ***ASCL1*** | **rs2291854** | **12** | **103353538** | **0.28(0.05)** | **0.02** | **1.33E-08** | **A/G** | 0.13 | **3'UTR** |
| Phe | *ASCL1* | rs17450122 | 12 | 103354394 | 0.41(0.06) | 0.04 | 2.51e-12 | A/G | 0.09 | Downstream |
| Phe | *ASCL1* | rs4578454 | 12 | 103354773 | 0.28(0.05) | 0.02 | 2.48e-08 | C/T | 0.13 | Downstream |
| Phe | *ASCL1* | rs1353769 | 12 | 103358503 | 0.27(0.05) | 0.02 | 3.02e-08 | G/T | 0.13 | Intergenic |
| Phe | *ASCL1* | rs36104021 | 12 | 103361112 | 0.37(0.06) | 0.03 | 1.60e-10 | C/G | 0.09 | Intergenic |
| **Phe** | ***ASCL1*** | **rs17450273** | **12** | **103361379** | **0.35(0.06)** | **0.03** | **6.60E-10** | **C/A** | 0.10 | **Intergenic** |
| Phe | *ASCL1* | rs17842959 | 12 | 103368889 | 0.34(0.06) | 0.03 | 2.20e-09 | A/T | 0.10 | Intergenic |
| Phe | *ASCL1* | rs4764719 | 12 | 103370585 | 0.29(0.05) | 0.02 | 1.15e-08 | A/C | 0.13 | Intergenic |
| Phe | *ASCL1* | rs6539060 | 12 | 103373017 | 0.34(0.06) | 0.03 | 2.89e-09 | G/A | 0.10 | Intergenic |
| Phe | *ASCL1* | rs7342307 | 12 | 103382325 | 0.34(0.06) | 0.03 | 1.55e-09 | C/T | 0.10 | Intergenic |
| Phe | *ASCL1* | rs1566299 | 12 | 103385509 | 0.28(0.05) | 0.02 | 1.91e-08 | C/G | 0.13 | Intergenic |
| Phe | *ASCL1* | rs35952479 | 12 | 103387965 | 0.34(0.06) | 0.03 | 1.45e-09 | A/G | 0.10 | Intergenic |
| Phe | *ASCL1* | rs2373952 | 12 | 103394540 | 0.33(0.06) | 0.03 | 2.52e-09 | A/G | 0.10 | Intergenic |
| Phe | *ASCL1* | rs28735715 | 12 | 103399186 | 0.34(0.06) | 0.03 | 2.68e-09 | A/T | 0.10 | Intergenic |
| Phe | *ASCL1* | rs3858690 | 12 | 103405397 | 0.34(0.06) | 0.03 | 2.36e-09 | C/T | 0.10 | Intergenic |
| Phe | *ASCL1* | rs78472299 | 12 | 103405697 | 0.36(0.06) | 0.03 | 4.75e-10 | T/C | 0.10 | Intergenic |
| Lys | *LOC646982* | rs9549153 | 13 | 40867548 | 0.22(0.04) | 0.02 | 2.79e-08 | G/A | 0.26 | Intergenic |
| Asn | *ASPG* | rs737394 | 14 | 104565922 | 0.81(0.05) | 0.15 | 1.19e-50 | G/T | 0.13 | Intronic |
| Asn | *ASPG* | rs1744297 | 14 | 104568472 | 0.82(0.05) | 0.16 | 1.30e-51 | T/C | 0.13 | Intronic |
| Asn | *ASPG* | rs2298000 | 14 | 104575326 | 0.77(0.05) | 0.15 | 1.03e-48 | C/A | 0.14 | Intronic |
| Asn | *ASPG* | rs34362765 | 14 | 104576448 | -0.24(0.04) | 0.03 | 1.59e-10 | G/A | 0.49 | Intronic |
| Asn | *MIR203* | rs61997624 | 14 | 104582386 | 0.78(0.05) | 0.14 | 2.69e-45 | C/T | 0.13 | Intergenic |
| Asn | *MIR203* | rs12589461 | 14 | 104588056 | 0.69(0.05) | 0.11 | 3.89e-37 | A/G | 0.13 | Intergenic |
| Asn | *KIF26A* | rs12587001 | 14 | 104602177 | 0.69(0.05) | 0.12 | 2.04e-37 | C/T | 0.13 | Intergenic |
| Ala | *DLGAP1* | rs588682 | 18 | 3558078 | 0.38(0.07) | 0.02 | 2.52e-08 | G/A | 0.09 | Intronic |
| Pro | *DGCR6* | rs116267673 | 22 | 18889967 | 0.45(0.07) | 0.03 | 2.63e-10 | C/A | 0.08 | Intergenic |
| Pro | *DGCR6* | rs201560606 | 22 | 18889969 | 0.45(0.07) | 0.03 | 2.63e-10 | G/A | 0.08 | Intergenic |
| Pro | *DGCR6* | rs2080346 | 22 | 18892575 | 0.26(0.04) | 0.03 | 2.62e-10 | G/A | 0.31 | Intergenic |
| Pro | *PRODH* | rs383964 | 22 | 18900669 | 0.56(0.10) | 0.02 | 9.86e-09 | A/G | 0.04 | 3'UTR |
| Pro | *PRODH* | rs450046 | 22 | 18901004 | 0.56(0.10) | 0.02 | 9.86e-09 | C/T | 0.04 | Exonic,Non-synonymous |
| Pro | *PRODH* | rs77193523 | 22 | 18905556 | 0.53(0.08) | 0.04 | 2.40e-12 | G/A | 0.06 | Intronic |
| Pro | *PRODH* | rs2277834 | 22 | 18910545 | 0.66(0.05) | 0.12 | 1.33e-37 | C/T | 0.15 | Intronic |
| Pro | *PRODH* | rs2238732 | 22 | 18915347 | 0.69(0.05) | 0.12 | 5.96e-40 | C/T | 0.14 | Intronic |
| Pro | *PRODH* | rs759404 | 22 | 18916180 | 0.68(0.06) | 0.09 | 3.49e-30 | C/T | 0.10 | Intronic |

The associations identified in genotyped data are highlighted as grey background and bold font.

Imputation was performed using the genotyping results of 665 samples that were unrelated to those used for the present study.

*a*; single nucleotide polymorphism, *b*; chromosome, *c*; standard error
