# Supplementary material for: Genetic basis for plasma amino acid concentrations based on absolute quantification: a genome-wide association study in the Japanese population
Source: Eur J Hum Genet. 2019 Jan 18;27(4):621–30. doi: 10.1038/s41431-018-0296-y (PMC6460579; doi:10.1038/s41431-018-0296-y)
Supplement: Supplementary file 2 — Supplementary table S2 [file 41431_2018_296_MOESM2_ESM.docx]

Supplementary table S2. The estimated regression models for each PFAA concentration used in GWAS-2.

| Models |
| --- |
| a-ABA ~ Arg +Asn +Gly +Ile +Leu +Lys +Met +Orn +Ser +Thr +Trp |
| Ala ~ a-ABA +Asn +Cit +Lys +Met +Orn +Pro +Trp +Tyr +Val |
| Arg ~ a-ABA +Asn +Cit +Gln +His +Ile +Leu +Lys +Orn +Thr +Tyr |
| Asn~ a-ABA +Arg +Gln +His +Met +Orn +Phe +Ser +Thr +Val |
| Cit ~ Ala +Arg +Glu +Lys +Orn +Phe +Pro |
| Gln ~ Arg +Glu +Gly +His +Leu +Orn +Ser +Val |
| Glu ~ Cit +Gln +Orn +Ser +Tyr +Val |
| Gly ~ a-ABA +Ala +Cit +Gln +His +Leu +Lys +Ser +Tyr |
| His ~ Ala +Asn +Gln +Gly +Ile +Leu +Lys +Phe +Ser |
| Ile ~ a-ABA +Arg +His +Leu +Lys +Met +Orn +Pro +Val |
| Leu ~ a-ABA +Arg +Gln +His +Ile +Lys +Phe +Pro +Ser +Thr +Val |
| Lys ~ a-ABA +Arg +Cit +Gly +His +Ile +Leu +Met +Orn +Phe +Ser +Trp +Tyr +Val |
| Met ~ a-ABA +Ala +Asn +Glu +Gly +Lys +Orn +Phe +Thr +Trp +Tyr |
| Orn ~ a-ABA +Ala +Arg +Asn +Cit +Gln +Glu +His +Lys +Met +Pro +Ser +Thr +Tyr |
| Phe ~ Asn +Cit +Gly +His +Leu +Lys +Met +Thr +Trp +Tyr |
| Pro ~ Ala +Cit +Ile +Leu +Thr |
| Ser ~ a-ABA +Asn +Cit +Gln +Glu +Gly +Lys +Orn +Thr +Trp |
| Thr ~ a-ABA +Arg +Asn +Glu +Leu +Met +Orn +Phe +Pro +Ser +Val |
| Trp ~ Ala +Lys +Met +Phe +Ser +Tyr |
| Tyr ~ Ala +Arg +Glu +Lys +Met +Orn +Phe +Trp +Val |
| Val ~ Ala +Asn +Gln +His +Ile +Leu +Lys +Met +Thr +Tyr |

Imputation was performed using the genotyping results of 665 samples that were unrelated to those used for the present study.
