# Supplementary material for: Genetic basis for plasma amino acid concentrations based on absolute quantification: a genome-wide association study in the Japanese population
Source: Eur J Hum Genet. 2019 Jan 18;27(4):621–30. doi: 10.1038/s41431-018-0296-y (PMC6460579; doi:10.1038/s41431-018-0296-y)
Supplement: Supplementary file 3 — Supplementary table S3 [file 41431_2018_296_MOESM3_ESM.docx]

Supplementary table S3. All the significant associations identified in GWAS-2.

| **Trait** | **Locus** | **SNP*^a^* (rs ID)** | **Chr*^b^*** | **Position** | **Beta (SE*^c^*)** | ***r*^2^** | ***P* value** | **Ref. (A1) /Var. (A2).** | **Freq. (A1)** | **Annotation** |
| --- | --- | --- | --- | --- | --- | --- | --- | --- | --- | --- |
| Gly | *CPS1* | rs34449727 | 2 | 211514995 | 0.31(0.05) | 0.02 | 6.90e-09 | G/T | 0.10 | Intronic |
| Gly | *CPS1* | rs4567871 | 2 | 211539302 | 0.23(0.03) | 0.03 | 6.57e-12 | C/T | 0.30 | Intronic |
| Gly | *CPS1* | rs715 | 2 | 211543055 | 0.72(0.04) | 0.22 | 1.83e-75 | T/C | 0.17 | 3'UTR |
| Gly | *CPS1* | rs4142152 | 2 | 211546043 | 0.35(0.03) | 0.09 | 4.14e-29 | C/T | 0.35 | Intergenic |
| Gly | *CPS1* | rs13386028 | 2 | 211547400 | 0.35(0.03) | 0.09 | 4.14e-29 | C/A | 0.35 | Intergenic |
| Gly | *CPS1* | rs4672588 | 2 | 211553159 | 0.34(0.03) | 0.09 | 1.15e-28 | T/C | 0.35 | Intergenic |
| Gly | *CPS1* | rs2887915 | 2 | 211554574 | 0.34(0.03) | 0.09 | 1.15e-28 | C/T | 0.35 | Intergenic |
| Gly | *CPS1* | rs10490320 | 2 | 211556975 | 0.35(0.03) | 0.09 | 5.23e-29 | T/C | 0.35 | Intergenic |
| Gly | *CPS1* | rs2371023 | 2 | 211559526 | 0.35(0.03) | 0.09 | 5.23e-29 | G/A | 0.35 | Intergenic |
| Gly | *CPS1* | rs79832648 | 2 | 211561489 | 0.34(0.03) | 0.09 | 1.09e-28 | C/T | 0.35 | Intergenic |
| Gly | *CPS1* | rs4673546 | 2 | 211565692 | 0.72(0.04) | 0.22 | 3.49e-74 | C/T | 0.16 | Intergenic |
| Gly | *CPS1* | rs12613336 | 2 | 211569399 | 0.72(0.04) | 0.22 | 9.35e-73 | T/C | 0.15 | Intergenic |
| Gly | *CPS1* | rs1016396 | 2 | 211570448 | 0.41(0.03) | 0.11 | 4.67e-36 | C/T | 0.28 | Intergenic |
| Gly | *CPS1* | rs4673547 | 2 | 211571520 | 0.42(0.03) | 0.12 | 2.98e-38 | A/G | 0.29 | Intergenic |
| Gly | *CPS1* | rs3815630 | 2 | 211581142 | 0.72(0.04) | 0.22 | 9.35e-73 | T/C | 0.15 | Intergenic |
| Gly | *CPS1* | rs7583500 | 2 | 211583066 | 0.42(0.03) | 0.11 | 5.10e-37 | T/C | 0.27 | Intergenic |
| Gly | *CPS1* | rs1861897 | 2 | 211585271 | 0.43(0.03) | 0.12 | 2.21e-40 | C/T | 0.28 | Intergenic |
| Gly | *CPS1* | rs12464097 | 2 | 211589248 | 0.43(0.03) | 0.12 | 3.07e-39 | A/C | 0.28 | Intergenic |
| Gly | *CPS1* | rs10932355 | 2 | 211600311 | 0.67(0.04) | 0.17 | 2.89e-55 | A/G | 0.14 | Intergenic |
| Gly | *CPS1* | rs2160847 | 2 | 211601116 | 0.67(0.04) | 0.17 | 1.98e-54 | T/C | 0.14 | Intergenic |
| Gly | *CPS1* | rs16844874 | 2 | 211602416 | 0.67(0.04) | 0.17 | 1.98e-54 | T/C | 0.14 | Intergenic |
| Gly | *CPS1* | rs10515953 | 2 | 211608583 | 0.38(0.03) | 0.10 | 2.13e-32 | G/A | 0.31 | Intergenic |
| Gly | *CPS1* | rs10206976 | 2 | 211614638 | 0.67(0.04) | 0.17 | 1.18e-54 | G/T | 0.14 | Intergenic |
| Gly | *CPS1* | rs4673554 | 2 | 211616067 | 0.38(0.03) | 0.10 | 3.57e-32 | G/T | 0.31 | Intergenic |
| Gly | *CPS1* | rs2287411 | 2 | 211618187 | 0.32(0.03) | 0.07 | 4.68e-22 | C/G | 0.28 | Intergenic |
| Gly | *CPS1* | rs11689207 | 2 | 211619571 | 0.58(0.09) | 0.03 | 1.83e-11 | A/G | 0.04 | Intergenic |
| Gly | *CPS1* | rs10490327 | 2 | 211619881 | 0.58(0.09) | 0.03 | 1.83e-11 | G/A | 0.04 | Intergenic |
| Gly | *CPS1* | rs2371074 | 2 | 211628462 | 0.38(0.03) | 0.10 | 5.94e-32 | C/T | 0.31 | Intergenic |
| Gly | *CPS1* | rs6736693 | 2 | 211629715 | 0.38(0.03) | 0.10 | 3.61e-32 | A/G | 0.31 | Intergenic |
| Gly | *CPS1* | rs10208743 | 2 | 211633505 | 0.38(0.03) | 0.10 | 1.73e-32 | A/G | 0.31 | Intergenic |
| Gly | *CPS1* | rs10201195 | 2 | 211637823 | 0.32(0.03) | 0.06 | 6.54e-21 | C/A | 0.27 | Intergenic |
| Gly | *CPS1* | rs969816 | 2 | 211643761 | 0.35(0.03) | 0.09 | 3.77e-28 | A/G | 0.34 | Intergenic |
| Gly | *CPS1* | rs10172053 | 2 | 211644647 | 0.64(0.04) | 0.15 | 2.44e-50 | T/G | 0.14 | Intergenic |
| Gly | *CPS1* | rs10932361 | 2 | 211647733 | 0.34(0.03) | 0.09 | 8.65e-28 | T/C | 0.34 | Intergenic |
| Gly | *CPS1* | rs4672596 | 2 | 211648806 | 0.34(0.03) | 0.09 | 6.03e-28 | T/C | 0.34 | Intergenic |
| Gly | *CPS1* | rs7568719 | 2 | 211653368 | 0.31(0.03) | 0.06 | 1.40e-20 | C/T | 0.28 | Intergenic |
| Gly | *CPS1* | rs6758081 | 2 | 211655965 | 0.26(0.04) | 0.04 | 6.18e-13 | A/G | 0.25 | Intergenic |
| Gly | *CPS1* | rs10205894 | 2 | 211657098 | 0.25(0.04) | 0.04 | 1.69e-12 | A/G | 0.24 | Intergenic |
| Gly | *CPS1* | rs4673558 | 2 | 211661897 | 0.43(0.04) | 0.08 | 1.15e-25 | C/T | 0.16 | Intergenic |
| Gly | *CPS1* | rs12998006 | 2 | 211669006 | 0.43(0.04) | 0.08 | 1.15e-25 | T/C | 0.16 | Intergenic |
| Gly | *CPS1* | rs16824978 | 2 | 211672061 | 0.25(0.04) | 0.04 | 3.37e-12 | C/T | 0.25 | Intergenic |
| Gly | *CPS1* | rs13012874 | 2 | 211673049 | 0.25(0.04) | 0.03 | 5.32e-12 | G/A | 0.24 | Intergenic |
| Gly | *CPS1* | rs36037305 | 2 | 211684149 | 0.30(0.04) | 0.04 | 3.57e-13 | A/G | 0.15 | Intergenic |
| Gly | *CPS1* | rs62202017 | 2 | 211697251 | 0.32(0.04) | 0.04 | 2.09e-14 | T/C | 0.15 | Intergenic |
| Gly | *CPS1* | rs13019409 | 2 | 211701811 | 0.31(0.04) | 0.04 | 2.41e-14 | A/G | 0.15 | Intergenic |
| Gly | *CPS1* | rs6726552 | 2 | 211740016 | 0.28(0.04) | 0.04 | 2.81e-13 | G/A | 0.20 | Intergenic |
| Gly | *CPS1* | rs6759308 | 2 | 211744633 | 0.27(0.04) | 0.04 | 7.44e-13 | G/T | 0.20 | Intergenic |
| Gly | *CPS1* | rs4310999 | 2 | 211750298 | 0.30(0.04) | 0.04 | 1.35e-12 | G/A | 0.14 | Intergenic |
| Gly | *CPS1* | rs12989482 | 2 | 211761183 | 0.19(0.03) | 0.02 | 8.14e-09 | T/C | 0.28 | Intergenic |
| Gly | *CPS1* | rs4327185 | 2 | 211764584 | 0.19(0.03) | 0.02 | 1.04e-08 | C/T | 0.27 | Intergenic |
| Gly | *CPS1* | rs6743162 | 2 | 211765062 | 0.19(0.03) | 0.02 | 1.04e-08 | C/T | 0.27 | Intergenic |
| Gly | *CPS1* | rs4338916 | 2 | 211766532 | 0.30(0.04) | 0.04 | 2.09e-12 | G/T | 0.14 | Intergenic |
| Gly | *CPS1* | rs12465522 | 2 | 211803160 | 0.27(0.04) | 0.03 | 1.55e-10 | T/G | 0.15 | Intergenic |
| Gly | *CPS1* | rs4455102 | 2 | 211808697 | 0.27(0.04) | 0.03 | 1.03e-10 | A/G | 0.15 | Intergenic |
| Gly | *RPS27P10* | rs523817 | 2 | 211856057 | 0.26(0.04) | 0.03 | 6.70e-10 | A/G | 0.14 | Intergenic |
| Gly | *RPS27P10* | rs651797 | 2 | 211856725 | 0.26(0.04) | 0.03 | 6.70e-10 | G/A | 0.14 | Intergenic |
| Gly | *RPS27P10* | rs200298971 | 2 | 211858883 | 0.26(0.04) | 0.03 | 6.70e-10 | G/T | 0.14 | Intergenic |
| Gly | *RPS27P10* | rs1657863 | 2 | 211859726 | 0.23(0.04) | 0.03 | 6.84e-10 | T/G | 0.21 | Intergenic |
| Gly | *RPS27P10* | rs1652312 | 2 | 211862218 | 0.26(0.04) | 0.03 | 6.70e-10 | T/C | 0.14 | Intergenic |
| Ser | *PSPHL* | rs11766051 | 7 | 55838545 | -0.18(0.02) | 0.04 | 1.03e-12 | G/T | 0.41 | NA |
| Ser | *PSPHL* | rs11976304 | 7 | 55840503 | -0.18(0.02) | 0.04 | 9.76e-13 | T/C | 0.41 | NA |
| Ser | *PSPHL* | rs6700 | 7 | 55841188 | -0.18(0.02) | 0.04 | 9.76e-13 | C/T | 0.41 | NA |
| Ser | *PSPHL* | rs6966061 | 7 | 55848790 | -0.18(0.02) | 0.04 | 9.01e-13 | A/G | 0.41 | Intergenic |
| Ser | *SEPT14* | rs11982736 | 7 | 55855180 | -0.18(0.02) | 0.04 | 2.25e-12 | G/A | 0.44 | Intergenic |
| Ser | *SEPT14* | rs9642404 | 7 | 55856799 | -0.18(0.02) | 0.04 | 5.36e-13 | G/A | 0.41 | Intergenic |
| Ser | *SEPT14* | rs28803557 | 7 | 55880211 | -0.18(0.02) | 0.04 | 9.00e-13 | G/A | 0.42 | Intronic |
| Ser | *SEPT14* | rs10233558 | 7 | 55881666 | -0.18(0.02) | 0.04 | 5.97e-13 | C/T | 0.42 | Intronic |
| Ser | *SEPT14* | rs4588807 | 7 | 55889109 | -0.19(0.02) | 0.04 | 3.61e-14 | A/G | 0.48 | Intronic |
| Ser | *SEPT14* | rs1113765 | 7 | 55889334 | -0.18(0.02) | 0.04 | 1.26e-13 | G/A | 0.47 | Intronic |
| Ser | *SEPT14* | rs10230845 | 7 | 55905896 | -0.19(0.02) | 0.04 | 3.97e-14 | C/T | 0.42 | Intronic |
| Ser | *SEPT14* | rs10239528 | 7 | 55908211 | -0.20(0.02) | 0.05 | 3.90e-15 | C/A | 0.46 | Intronic |
| Ser | *SEPT14* | rs4476962 | 7 | 55919938 | 0.17(0.03) | 0.03 | 1.14e-10 | C/T | 0.34 | Intronic |
| Ser | *SEPT14* | rs13233754 | 7 | 55941077 | 0.20(0.03) | 0.03 | 7.30e-12 | G/A | 0.26 | Intergenic |
| Ser | *SEPT14* | rs6593287 | 7 | 55944185 | -0.21(0.02) | 0.05 | 3.77e-18 | G/A | 0.47 | Intergenic |
| Ser | *SEPT14* | rs11761352 | 7 | 55950947 | -0.23(0.02) | 0.06 | 4.13e-19 | C/A | 0.39 | Intergenic |
| Ser | *SEPT14* | rs6947698 | 7 | 55954993 | -0.22(0.02) | 0.06 | 9.44e-19 | G/C | 0.48 | Intergenic |
| Ser | *ZNF713* | rs10271662 | 7 | 55962907 | -0.25(0.03) | 0.07 | 2.19e-22 | A/C | 0.36 | Intergenic |
| Ser | *ZNF713* | rs35304280 | 7 | 55972857 | 0.21(0.03) | 0.03 | 7.00e-12 | A/C | 0.22 | Intergenic |
| Ser | *ZNF713* | rs2135116 | 7 | 55991144 | -0.25(0.03) | 0.07 | 1.04e-22 | A/G | 0.36 | Intronic |
| Ser | *ZNF713* | rs13222366 | 7 | 55999702 | -0.23(0.02) | 0.06 | 3.18e-20 | G/A | 0.49 | Intronic |
| Ser | *MRPS17* | rs12671091 | 7 | 56018666 | -0.23(0.02) | 0.06 | 3.99e-20 | T/C | 0.48 | Upstream |
| Ser | *GBAS* | rs11238386 | 7 | 56033141 | 0.16(0.03) | 0.03 | 4.31e-09 | A/C | 0.33 | Intronic |
| Ser | *GBAS* | rs34929372 | 7 | 56034559 | 0.16(0.03) | 0.03 | 1.64e-09 | T/C | 0.33 | Intronic |
| Ser | *GBAS* | rs4360246 | 7 | 56036606 | 0.19(0.03) | 0.03 | 4.98e-11 | G/C | 0.24 | Intronic |
| Ser | *GBAS* | rs4535700 | 7 | 56045448 | -0.25(0.03) | 0.07 | 5.31e-22 | T/C | 0.37 | Intronic |
| Ser | *GBAS* | rs73343719 | 7 | 56046421 | 0.19(0.03) | 0.03 | 5.43e-11 | A/G | 0.24 | Intronic |
| Ser | *GBAS* | rs4543497 | 7 | 56047215 | -0.25(0.03) | 0.07 | 6.64e-22 | T/C | 0.37 | Intronic |
| Ser | *GBAS* | rs13239795 | 7 | 56055327 | 0.20(0.03) | 0.03 | 2.63e-11 | C/T | 0.21 | Intronic |
| Ser | *GBAS* | rs13245637 | 7 | 56068980 | 0.17(0.03) | 0.03 | 4.72e-10 | C/T | 0.34 | Intergenic |
| Ser | *PSPH* | rs35210477 | 7 | 56112217 | 0.21(0.03) | 0.03 | 7.41e-12 | A/G | 0.20 | Intronic |
| Ser | *PSPH* | rs6593296 | 7 | 56118293 | 0.20(0.03) | 0.03 | 2.11e-11 | C/T | 0.25 | Intronic |
| Ser | *CCT6A* | rs7793921 | 7 | 56120881 | -0.26(0.02) | 0.08 | 1.14e-24 | G/A | 0.39 | Intronic |
| Ser | *SUMF2* | rs13238899 | 7 | 56140618 | 0.17(0.03) | 0.03 | 1.95e-09 | T/G | 0.26 | Intronic |
| Ser | *SUMF2* | rs13244654 | 7 | 56146956 | -0.28(0.03) | 0.08 | 1.33e-25 | T/C | 0.33 | Intronic |
| Ser | *PHKG1* | rs2242508 | 7 | 56151489 | -0.28(0.03) | 0.08 | 1.39e-25 | A/G | 0.33 | Intronic |
| Ser | *CHCHD2* | rs4948106 | 7 | 56167595 | -0.24(0.02) | 0.06 | 5.25e-21 | T/G | 0.38 | Intergenic |
| Ser | *CHCHD2* | rs816411 | 7 | 56171489 | -0.24(0.02) | 0.06 | 7.06e-21 | C/T | 0.38 | Intronic |
| Ser | *CHCHD2* | rs35557048 | 7 | 56176832 | 0.20(0.03) | 0.03 | 1.52e-10 | G/A | 0.21 | Intergenic |
| Ser | *LOC389493* | rs7782243 | 7 | 56181845 | -0.20(0.03) | 0.04 | 2.53e-13 | A/G | 0.28 | Upstream |
| Ser | *LOC442309* | rs2538054 | 7 | 56210441 | -0.23(0.03) | 0.04 | 7.52e-14 | A/G | 0.22 | Intergenic |
| Ser | *LOC442309* | rs34148685 | 7 | 56223739 | 0.18(0.03) | 0.02 | 2.33e-08 | T/C | 0.20 | Intergenic |
| Ser | *LOC100130909* | rs816396 | 7 | 56237280 | -0.21(0.03) | 0.03 | 2.25e-11 | T/C | 0.20 | Intergenic |
| Arg | *SLC7A2* | rs56335308 | 8 | 17419461 | -0.43(0.05) | 0.05 | 2.64e-16 | G/A | 0.06 | Exonic,Non-synonymous |
| Orn | *SLC7A2* | rs56335308 | 8 | 17419461 | -0.35(0.05) | 0.04 | 4.70e-14 | G/A | 0.06 | Exonic,Non-synonymous |
| Arg | *SLC7A2* | rs55755124 | 8 | 17422481 | -0.43(0.05) | 0.05 | 7.63e-16 | T/C | 0.06 | Exonic,  Synonymous |
| Orn | *SLC7A2* | rs55755124 | 8 | 17422481 | -0.35(0.05) | 0.04 | 6.25e-14 | T/C | 0.06 | Exonic,  Synonymous |
| Arg | *SLC7A2* | rs7838611 | 8 | 17422717 | -0.43(0.05) | 0.05 | 1.62e-15 | A/G | 0.06 | 3'UTR |
| Orn | *SLC7A2* | rs7838611 | 8 | 17422717 | -0.35(0.05) | 0.04 | 7.61e-14 | A/G | 0.06 | 3'UTR |
| Arg | *SLC7A2* | rs7842106 | 8 | 17422818 | -0.43(0.05) | 0.05 | 1.62e-15 | A/G | 0.06 | 3'UTR |
| Orn | *SLC7A2* | rs7842106 | 8 | 17422818 | -0.35(0.05) | 0.04 | 7.61e-14 | A/G | 0.06 | 3'UTR |
| Arg | *SLC7A2* | rs7842267 | 8 | 17422901 | -0.43(0.05) | 0.05 | 1.62e-15 | A/G | 0.06 | 3'UTR |
| Orn | *SLC7A2* | rs7842267 | 8 | 17422901 | -0.35(0.05) | 0.04 | 7.61e-14 | A/G | 0.06 | 3'UTR |
| Arg | *SLC7A2* | rs7842456 | 8 | 17423065 | -0.43(0.05) | 0.05 | 1.62e-15 | A/G | 0.06 | 3'UTR |
| Orn | *SLC7A2* | rs7842456 | 8 | 17423065 | -0.35(0.05) | 0.04 | 7.61e-14 | A/G | 0.06 | 3'UTR |
| Arg | *SLC7A2* | rs7823792 | 8 | 17423171 | -0.43(0.05) | 0.05 | 1.62e-15 | G/A | 0.06 | 3'UTR |
| Orn | *SLC7A2* | rs7823792 | 8 | 17423171 | -0.35(0.05) | 0.04 | 7.61e-14 | G/A | 0.06 | 3'UTR |
| Arg | *SLC7A2* | rs74844803 | 8 | 17423433 | -0.43(0.05) | 0.05 | 1.62e-15 | C/T | 0.06 | 3'UTR |
| Orn | *SLC7A2* | rs74844803 | 8 | 17423433 | -0.35(0.05) | 0.04 | 7.61e-14 | C/T | 0.06 | 3'UTR |
| Arg | *SLC7A2* | rs6586610 | 8 | 17423513 | -0.27(0.05) | 0.03 | 5.17e-09 | G/T | 0.10 | 3'UTR |
| Orn | *SLC7A2* | rs6586610 | 8 | 17423513 | -0.23(0.04) | 0.02 | 9.28e-09 | G/T | 0.10 | 3'UTR |
| Arg | *SLC7A2* | rs7014645 | 8 | 17423656 | -0.43(0.05) | 0.05 | 1.62e-15 | A/G | 0.06 | 3'UTR |
| Orn | *SLC7A2* | rs7014645 | 8 | 17423656 | -0.35(0.05) | 0.04 | 7.61e-14 | A/G | 0.06 | 3'UTR |
| Arg | *SLC7A2* | rs6998033 | 8 | 17423856 | -0.43(0.05) | 0.05 | 1.62e-15 | G/T | 0.06 | 3'UTR |
| Orn | *SLC7A2* | rs6998033 | 8 | 17423856 | -0.35(0.05) | 0.04 | 7.61e-14 | G/T | 0.06 | 3'UTR |
| Arg | *SLC7A2* | rs6998037 | 8 | 17423866 | -0.43(0.05) | 0.05 | 1.62e-15 | G/A | 0.06 | 3'UTR |
| Orn | *SLC7A2* | rs6998037 | 8 | 17423866 | -0.35(0.05) | 0.04 | 7.61e-14 | G/A | 0.06 | 3'UTR |
| Arg | *SLC7A2* | rs6980866 | 8 | 17423942 | -0.43(0.05) | 0.05 | 1.62e-15 | A/G | 0.06 | 3'UTR |
| Orn | *SLC7A2* | rs6980866 | 8 | 17423942 | -0.35(0.05) | 0.04 | 7.61e-14 | A/G | 0.06 | 3'UTR |
| Arg | *SLC7A2* | rs2285295 | 8 | 17427541 | -0.35(0.04) | 0.04 | 1.67e-14 | A/G | 0.09 | 3'UTR |
| Orn | *SLC7A2* | rs2285295 | 8 | 17427541 | -0.30(0.04) | 0.04 | 1.28e-13 | A/G | 0.09 | 3'UTR |
| Arg | *SLC7A2* | rs7002951 | 8 | 17428276 | -0.34(0.04) | 0.04 | 3.23e-14 | A/G | 0.09 | Downstream |
| Orn | *SLC7A2* | rs7002951 | 8 | 17428276 | -0.29(0.04) | 0.04 | 2.46e-13 | A/G | 0.09 | Downstream |
| Orn | *PDGFRL* | rs7008481 | 8 | 17431678 | -0.18(0.03) | 0.02 | 2.17e-08 | G/A | 0.15 | Intergenic |
| Arg | *PDGFRL* | rs9792282 | 8 | 17438439 | -0.18(0.03) | 0.02 | 2.81e-08 | G/A | 0.21 | Intronic |
| Arg | *PDGFRL* | rs71526138 | 8 | 17438936 | -0.18(0.03) | 0.02 | 2.81e-08 | G/A | 0.21 | Intronic |
| Gln | *SPRYD4* | rs7302925 | 12 | 56861458 | -0.41(0.05) | 0.06 | 2.09e-18 | A/G | 0.09 | Upstream |
| Gln | *GLS2* | rs2657879 | 12 | 56865338 | -0.41(0.05) | 0.06 | 2.09e-18 | A/G | 0.09 | Exonic,Non-synonymous |
| Gln | *RBMS2* | rs774211 | 12 | 56920939 | -0.30(0.04) | 0.04 | 3.86e-13 | T/C | 0.12 | Intronic |
| Gln | *RBMS2* | rs2638301 | 12 | 56928814 | -0.24(0.04) | 0.03 | 1.12e-09 | A/G | 0.14 | Intronic |
| Gln | *RBMS2* | rs55961843 | 12 | 56956213 | -0.22(0.04) | 0.02 | 1.13e-08 | T/C | 0.15 | Exonic,  Synonymous |
| Gln | *RBMS2* | rs12232026 | 12 | 56960766 | -0.22(0.04) | 0.02 | 1.91e-08 | T/A | 0.14 | Intronic |
| Phe | *PAH* | rs772897 | 12 | 103237468 | 0.26(0.03) | 0.04 | 2.37e-14 | G/C | 0.14 | Exonic,  Synonymous |
| Phe | *PAH* | rs772893 | 12 | 103239609 | 0.27(0.03) | 0.05 | 3.98e-15 | T/C | 0.14 | Intronic |
| Phe | *PAH* | rs1722387 | 12 | 103241070 | 0.27(0.03) | 0.04 | 5.65e-15 | T/C | 0.14 | Intronic |
| Phe | *PAH* | rs2251897 | 12 | 103249327 | 0.27(0.03) | 0.05 | 3.40e-15 | G/A | 0.14 | Intronic |
| Phe | *PAH* | rs1718302 | 12 | 103272686 | 0.30(0.03) | 0.06 | 1.28e-18 | G/A | 0.14 | Intronic |
| Phe | *PAH* | rs10860936 | 12 | 103282952 | 0.27(0.04) | 0.03 | 4.32e-10 | T/C | 0.10 | Intronic |
| Phe | *PAH* | rs80238951 | 12 | 103291883 | 0.26(0.04) | 0.03 | 3.09e-09 | C/T | 0.10 | Intronic |
| Phe | *PAH* | rs78985461 | 12 | 103295636 | 0.34(0.04) | 0.06 | 8.05e-21 | G/A | 0.12 | Intronic |
| Phe | *PAH* | rs75236535 | 12 | 103300962 | 0.32(0.05) | 0.03 | 1.15e-11 | T/C | 0.09 | Intronic |
| Phe | *PAH* | rs34281125 | 12 | 103317416 | 0.31(0.03) | 0.05 | 4.20e-18 | C/T | 0.13 | Intergenic |
| Phe | *PAH* | rs71466246 | 12 | 103318463 | 0.31(0.03) | 0.06 | 2.01e-18 | G/A | 0.13 | Intergenic |
| Phe | *PAH* | rs34732688 | 12 | 103318823 | 0.28(0.03) | 0.05 | 4.18e-17 | A/G | 0.14 | Intergenic |
| Phe | *PAH* | rs17547025 | 12 | 103326270 | 0.28(0.03) | 0.05 | 4.18e-17 | A/C | 0.14 | Intergenic |
| Phe | *ASCL1* | rs34683293 | 12 | 103342743 | 0.29(0.04) | 0.05 | 5.14e-16 | A/T | 0.10 | Intergenic |
| Phe | *ASCL1* | rs7973625 | 12 | 103347511 | 0.40(0.04) | 0.06 | 3.10e-21 | T/C | 0.09 | Intergenic |
| Phe | *ASCL1* | rs2291854 | 12 | 103353538 | 0.27(0.03) | 0.05 | 1.53e-15 | A/G | 0.12 | 3'UTR |
| Phe | *ASCL1* | rs17450122 | 12 | 103354394 | 0.42(0.04) | 0.08 | 3.01e-25 | A/G | 0.09 | Downstream |
| Phe | *ASCL1* | rs4578454 | 12 | 103354773 | 0.27(0.03) | 0.05 | 7.56e-16 | C/T | 0.13 | Downstream |
| Phe | *ASCL1* | rs1353769 | 12 | 103358503 | 0.27(0.03) | 0.04 | 9.29e-15 | G/T | 0.13 | Intergenic |
| Phe | *ASCL1* | rs36104021 | 12 | 103361112 | 0.40(0.04) | 0.07 | 1.52e-23 | C/G | 0.09 | Intergenic |
| Phe | *ASCL1* | rs17450273 | 12 | 103361379 | 0.39(0.04) | 0.07 | 4.00e-23 | C/A | 0.10 | Intergenic |
| Phe | *ASCL1* | rs17842959 | 12 | 103368889 | 0.35(0.04) | 0.06 | 7.73e-20 | A/T | 0.10 | Intergenic |
| Phe | *ASCL1* | rs4764719 | 12 | 103370585 | 0.27(0.03) | 0.04 | 1.53e-14 | A/C | 0.13 | Intergenic |
| Phe | *ASCL1* | rs6539060 | 12 | 103373017 | 0.35(0.04) | 0.06 | 1.72e-19 | G/A | 0.10 | Intergenic |
| Phe | *ASCL1* | rs7342307 | 12 | 103382325 | 0.35(0.04) | 0.06 | 1.03e-19 | C/T | 0.10 | Intergenic |
| Phe | *ASCL1* | rs1566299 | 12 | 103385509 | 0.25(0.03) | 0.04 | 1.16e-13 | C/G | 0.13 | Intergenic |
| Phe | *ASCL1* | rs882461 | 12 | 103387712 | 0.21(0.03) | 0.04 | 4.02e-12 | T/C | 0.18 | Intergenic |
| Phe | *ASCL1* | rs35952479 | 12 | 103387965 | 0.34(0.04) | 0.06 | 7.74e-19 | A/G | 0.10 | Intergenic |
| Phe | *ASCL1* | rs2373952 | 12 | 103394540 | 0.34(0.04) | 0.06 | 6.82e-19 | A/G | 0.10 | Intergenic |
| Phe | *ASCL1* | rs67399100 | 12 | 103398582 | 0.25(0.03) | 0.04 | 1.78e-13 | T/C | 0.13 | Intergenic |
| Phe | *ASCL1* | rs28735715 | 12 | 103399186 | 0.35(0.04) | 0.06 | 2.45e-19 | A/T | 0.10 | Intergenic |
| Phe | *ASCL1* | rs7962773 | 12 | 103401458 | 0.25(0.03) | 0.04 | 2.67e-13 | G/T | 0.13 | Intergenic |
| Phe | *ASCL1* | rs3858690 | 12 | 103405397 | 0.35(0.04) | 0.06 | 3.63e-19 | C/T | 0.10 | Intergenic |
| Phe | *ASCL1* | rs78472299 | 12 | 103405697 | 0.36(0.04) | 0.06 | 8.07e-20 | T/C | 0.10 | Intergenic |
| Phe | *ASCL1* | rs34207056 | 12 | 103421047 | 0.21(0.03) | 0.03 | 2.75e-10 | C/T | 0.13 | Intergenic |
| Asn | *TDRD9* | rs33984773 | 14 | 104393766 | 0.15(0.03) | 0.02 | 1.56e-08 | G/A | 0.32 | Intergenic |
| Asn | *ASPG* | rs737394 | 14 | 104565922 | 0.77(0.03) | 0.30 | 6.78e-106 | G/T | 0.13 | Intronic |
| Asn | *ASPG* | rs1744297 | 14 | 104568472 | 0.77(0.03) | 0.30 | 3.30e-107 | T/C | 0.13 | Intronic |
| Asn | *ASPG* | rs2298000 | 14 | 104575326 | 0.72(0.03) | 0.29 | 2.17e-100 | C/A | 0.14 | Intronic |
| Asn | *ASPG* | rs34362765 | 14 | 104576448 | -0.25(0.03) | 0.07 | 1.74e-22 | G/A | 0.49 | Intronic |
| Asn | *MIR203* | rs61997624 | 14 | 104582386 | 0.73(0.03) | 0.27 | 1.50e-91 | C/T | 0.13 | Intergenic |
| Asn | *MIR203* | rs61997625 | 14 | 104585263 | -0.21(0.03) | 0.05 | 2.52e-16 | C/T | 0.46 | Intergenic |
| Asn | *MIR203* | rs12588869 | 14 | 104587348 | -0.21(0.03) | 0.05 | 5.84e-16 | G/A | 0.50 | Intergenic |
| Asn | *MIR203* | rs12589461 | 14 | 104588056 | 0.65(0.03) | 0.22 | 1.91e-73 | A/G | 0.13 | Intergenic |
| Asn | *KIF26A* | rs77730480 | 14 | 104599420 | 0.64(0.11) | 0.02 | 3.01e-08 | C/T | 0.02 | Intergenic |
| Asn | *KIF26A* | rs12587001 | 14 | 104602177 | 0.65(0.03) | 0.22 | 4.88e-74 | C/T | 0.13 | Intergenic |
| Asn | *KIF26A* | rs11628529 | 14 | 104607281 | 0.44(0.07) | 0.03 | 2.90e-09 | G/A | 0.04 | Intronic |
| Asn | *KIF26A* | rs2011069 | 14 | 104608068 | -0.18(0.03) | 0.04 | 1.59e-12 | A/G | 0.49 | Intronic |
| Asn | *KIF26A* | rs12886129 | 14 | 104609413 | 0.42(0.07) | 0.02 | 9.93e-09 | T/C | 0.04 | Intronic |
| Asn | *KIF26A* | rs4906421 | 14 | 104613478 | -0.18(0.03) | 0.04 | 1.55e-12 | A/G | 0.49 | Intronic |
| Asn | *KIF26A* | rs8019366 | 14 | 104616226 | 0.14(0.03) | 0.02 | 1.90e-08 | A/G | 0.43 | Intronic |
| Asn | *KIF26A* | rs12588775 | 14 | 104617095 | 0.43(0.07) | 0.02 | 1.47e-08 | C/T | 0.04 | Intronic |
| Asn | *KIF26A* | rs12147202 | 14 | 104620678 | 0.20(0.03) | 0.04 | 1.06e-14 | A/C | 0.48 | Intronic |
| Gly | *PKD1L2* | rs4888121 | 16 | 81143509 | -0.18(0.03) | 0.02 | 2.55e-08 | T/C | 0.29 | Intronic |
| Gly | *PKD1L2* | rs8059153 | 16 | 81145675 | -0.21(0.04) | 0.02 | 1.46e-08 | T/C | 0.20 | Intronic |
| Gly | *PKD1L2* | rs4258631 | 16 | 81147730 | -0.21(0.04) | 0.02 | 2.62e-08 | C/T | 0.20 | Intronic |
| Pro | *DGCR6* | rs116267673 | 22 | 18889967 | 0.40(0.06) | 0.04 | 1.58e-12 | C/A | 0.08 | Intergenic |
| Pro | *DGCR6* | rs201560606 | 22 | 18889969 | 0.40(0.06) | 0.04 | 1.58e-12 | G/A | 0.08 | Intergenic |
| Pro | *DGCR6* | rs2080346 | 22 | 18892575 | 0.21(0.03) | 0.03 | 1.54e-10 | G/A | 0.31 | Intergenic |
| Pro | *PRODH* | rs383964 | 22 | 18900669 | 0.52(0.08) | 0.03 | 5.00e-11 | A/G | 0.04 | 3'UTR |
| Pro | *PRODH* | rs450046 | 22 | 18901004 | 0.52(0.08) | 0.03 | 5.00e-11 | C/T | 0.04 | Exonic,Non-synonymous |
| Pro | *PRODH* | rs77193523 | 22 | 18905556 | 0.52(0.06) | 0.05 | 1.07e-17 | G/A | 0.06 | Intronic |
| Ala | *PRODH* | rs2277834 | 22 | 18910545 | -0.24(0.04) | 0.03 | 2.89e-10 | C/T | 0.15 | Intronic |
| Pro | *PRODH* | rs2277834 | 22 | 18910545 | 0.70(0.04) | 0.20 | 9.54e-67 | C/T | 0.15 | Intronic |
| Ala | *PRODH* | rs2238732 | 22 | 18915347 | -0.25(0.04) | 0.03 | 7.10e-11 | C/T | 0.14 | Intronic |
| Pro | *PRODH* | rs2238732 | 22 | 18915347 | 0.72(0.04) | 0.21 | 9.88e-70 | C/T | 0.14 | Intronic |
| Ala | *PRODH* | rs759404 | 22 | 18916180 | -0.25(0.04) | 0.03 | 5.10e-09 | C/T | 0.10 | Intronic |
| Pro | *PRODH* | rs759404 | 22 | 18916180 | 0.73(0.04) | 0.17 | 4.53e-55 | C/T | 0.10 | Intronic |

Imputation was performed using the genotyping results of 665 samples that were unrelated to those used for the present study.

*a*; single nucleotide polymorphism, *b*; chromosome, *c*; standard error
