# Supplementary material for: Genetic basis for plasma amino acid concentrations based on absolute quantification: a genome-wide association study in the Japanese population
Source: Eur J Hum Genet. 2019 Jan 18;27(4):621–30. doi: 10.1038/s41431-018-0296-y (PMC6460579; doi:10.1038/s41431-018-0296-y)
Supplement: Supplementary file 4 — Supplementary table S4 [file 41431_2018_296_MOESM4_ESM.docx]

Supplementary table S4. *P* values of each locus significantly associated with PFAA concentrations conditioned on multiple PFAAs or one of the six PFAAs.

| Locus | SNP*^a^* | GWAS- | Covariates in GWAS-3 | Ala | a-ABA | Arg | Asn | Cit | Gln | Glu | Gly | His | Ile | Met | Leu | Lys | Orn | Phe | Pro | Ser | Thr | Trp | Tyr | Val |
| --- | --- | --- | --- | --- | --- | --- | --- | --- | --- | --- | --- | --- | --- | --- | --- | --- | --- | --- | --- | --- | --- | --- | --- | --- |
| *CPS1* | rs12613336 | |  |  |  |  |  |  |  |  |  |  |  |  |  |  |  |  |  |  |  |  |  |  |
|  |  | 1 |  | 3.32e-01 | 8.18e-01 | 1.02e-04 | 2.71e-01 | 1.29e-01 | 4.12e-01 | 3.58e-01 | **2.07e-70** | 1.05e-03 | 8.72e-01 | 5.93e-01 | 8.96e-01 | 6.05e-01 | 3.20e-01 | 1.67e-02 | 1.09e-01 | **4.77e-12** | 2.16e-01 | 1.98e-01 | 6.39e-01 | 8.76e-01 |
|  |  | 2 |  | 6.91e-01 | 5.61e-01 | 1.01e-06 | 4.23e-01 | 4.67e-01 | 1.41e-01 | 9.07e-01 | **9.35e-73** | 7.67e-01 | 8.97e-01 | 5.46e-01 | 8.41e-01 | 7.77e-01 | 8.58e-04 | 1.57e-01 | 2.81e-01 | 6.29e-01 | 2.85e-03 | 6.49e-01 | 2.49e-01 | 1.51e-01 |
|  |  | 3 | Asn | 5.59e-01 | 6.69e-01 | 1.72e-04 | N.T.*^b^* | 2.08e-01 | 1.40e-01 | 3.55e-01 | **2.47e-82** | 1.77e-03 | 8.95e-01 | 1.29e-01 | 6.69e-01 | 9.80e-01 | 5.46e-01 | 2.98e-02 | 1.75e-01 | **4.56e-17** | 4.90e-01 | 3.14e-01 | 9.62e-01 | 6.73e-01 |
|  |  |  | Gln | 1.99e-01 | 9.07e-01 | 1.09e-05 | 1.06e-01 | 6.23e-02 | N.T.*^b^* | 4.02e-01 | **2.16e-80** | 1.22e-04 | 6.97e-01 | 8.47e-01 | 9.09e-01 | 3.40e-01 | 1.41e-01 | 7.78e-03 | 5.55e-02 | **3.84e-13** | 8.61e-02 | 1.43e-01 | 4.30e-01 | 9.86e-01 |
|  |  |  | Gly | 2.70e-05 | 8.60e-01 | **1.49e-13** | **2.09e-10** | 1.63e-07 | **1.57e-09** | 1.64e-01 | N.T.*^b^* | 8.53e-05 | 7.89e-01 | 1.67e-05 | 9.49e-01 | 8.93e-06 | **3.44e-09** | 1.69e-02 | 1.68e-05 | 2.06e-03 | **5.58e-11** | 8.80e-02 | 3.37e-02 | 6.12e-01 |
|  |  |  | Phe | 9.12e-01 | 4.38e-01 | 3.86e-04 | 7.12e-01 | 2.77e-01 | 1.55e-01 | 5.61e-01 | **2.09e-70** | 1.16e-02 | 2.19e-01 | 4.90e-02 | 7.11e-02 | 7.53e-01 | 7.56e-01 | N.T.*^b^* | 3.29e-01 | **2.12e-13** | 4.97e-01 | 8.25e-01 | 2.70e-01 | 1.19e-01 |
|  |  |  | Pro | 8.78e-01 | 6.77e-01 | 3.19e-04 | 4.99e-01 | 2.50e-01 | 1.74e-01 | 4.78e-01 | **4.02e-75** | 3.19e-03 | 5.45e-01 | 2.18e-01 | 4.58e-01 | 9.52e-01 | 6.43e-01 | 4.57e-02 | N.T.*^b^* | **8.68e-14** | 5.03e-01 | 3.46e-01 | 8.30e-01 | 3.87e-01 |
|  |  |  | Ser | 1.27e-02 | 5.78e-02 | 5.03e-08 | 4.08e-06 | 6.41e-03 | 2.11e-02 | 7.37e-01 | **4.45e-61** | 9.83e-06 | 2.69e-01 | 7.35e-03 | 3.14e-01 | 8.97e-03 | 4.24e-04 | 7.17e-04 | 2.47e-03 | N.T.*^b^* | 5.48e-08 | 7.15e-02 | 2.72e-02 | 5.05e-01 |
| *PSPH* | rs13244654 | |  |  |  |  |  |  |  |  |  |  |  |  |  |  |  |  |  |  |  |  |  |  |
|  |  | 1 |  | 3.82e-01 | 9.14e-01 | 6.85e-01 | 5.00e-01 | 1.13e-01 | 1.20e-01 | 2.54e-01 | 3.51e-04 | 5.83e-01 | 3.46e-01 | 5.50e-01 | 3.21e-01 | 4.11e-01 | 7.44e-01 | 4.61e-01 | 9.02e-01 | **1.80e-21** | 9.48e-01 | 1.21e-01 | 6.54e-01 | 5.72e-01 |
|  |  | 2 |  | 1.50e-01 | 1.72e-02 | 7.94e-01 | 5.15e-01 | 6.01e-02 | 4.28e-01 | 9.32e-01 | 3.03e-01 | 9.88e-01 | 2.82e-01 | 8.93e-02 | 2.84e-01 | 9.26e-01 | 1.33e-01 | 9.37e-02 | 7.53e-01 | **1.33e-25** | 2.35e-01 | 9.04e-01 | 7.64e-01 | 8.33e-03 |
|  |  | 3 | Asn | 5.71e-01 | 8.92e-01 | 4.90e-01 | N.T.*^b^* | 7.34e-02 | 1.69e-01 | 2.56e-01 | 4.35e-04 | 3.12e-01 | 4.38e-01 | 1.68e-01 | 3.94e-01 | 1.82e-01 | 4.62e-01 | 5.64e-01 | 8.55e-01 | **1.07e-24** | 5.37e-01 | 6.10e-02 | 4.03e-01 | 4.35e-01 |
|  |  |  | Gln | 7.43e-01 | 8.10e-01 | 3.48e-01 | 9.82e-01 | 3.55e-02 | N.T.*^b^* | 3.22e-01 | 1.26e-03 | 2.05e-01 | 5.88e-01 | 1.26e-01 | 5.72e-01 | 8.42e-02 | 2.52e-01 | 6.78e-01 | 6.65e-01 | **3.53e-22** | 4.44e-01 | 5.39e-02 | 2.74e-01 | 3.37e-01 |
|  |  |  | Gly | 9.15e-01 | 9.12e-01 | 2.66e-01 | 6.73e-01 | 1.92e-02 | 8.44e-01 | 5.07e-01 | N.T.*^b^* | 4.49e-01 | 3.77e-01 | 7.78e-02 | 3.35e-01 | 7.81e-02 | 1.47e-01 | 4.56e-01 | 5.75e-01 | **2.73e-18** | 2.37e-01 | 8.86e-02 | 3.81e-01 | 5.90e-01 |
|  |  |  | Phe | 5.95e-01 | 8.47e-01 | 5.96e-01 | 6.77e-01 | 8.55e-02 | 1.65e-01 | 2.04e-01 | 4.29e-04 | 3.03e-01 | 5.39e-01 | 2.12e-01 | 5.00e-01 | 2.12e-01 | 5.05e-01 | N.T.*^b^* | 8.26e-01 | **6.64e-21** | 7.84e-01 | 2.89e-02 | 2.39e-01 | 2.55e-01 |
|  |  |  | Pro | 3.74e-01 | 9.80e-01 | 6.79e-01 | 5.15e-01 | 1.13e-01 | 1.12e-01 | 2.49e-01 | 3.54e-04 | 5.24e-01 | 3.16e-01 | 4.44e-01 | 2.97e-01 | 3.38e-01 | 6.55e-01 | 4.37e-01 | N.T.*^b^* | **5.09e-22** | 9.28e-01 | 9.93e-02 | 5.68e-01 | 5.07e-01 |
|  |  |  | Ser | 2.18e-01 | 3.53e-03 | 1.76e-02 | 1.26e-04 | 1.59e-03 | 7.79e-03 | 7.25e-01 | 1.55e-01 | 3.53e-02 | 7.23e-01 | 1.67e-07 | 5.85e-01 | 1.64e-04 | 1.65e-04 | 5.83e-01 | 6.21e-02 | N.T.*^b^* | **3.82e-08** | 2.09e-02 | 4.07e-03 | 8.31e-02 |
| *GLS2* | rs7302925 |  |  |  |  |  |  |  |  |  |  |  |  |  |  |  |  |  |  |  |  |  |  |  |
|  |  | 1 |  | 9.68e-01 | 4.60e-02 | 2.97e-01 | 9.93e-01 | 4.24e-02 | **9.73e-11** | 4.98e-01 | 7.59e-01 | 5.11e-01 | 1.31e-01 | 8.53e-01 | 6.02e-01 | 9.82e-01 | 7.79e-01 | 4.83e-01 | 3.34e-01 | 7.85e-01 | 3.69e-01 | 2.85e-01 | 9.52e-01 | 7.05e-01 |
|  |  | 2 |  | 7.90e-01 | 6.15e-02 | 3.04e-01 | 9.49e-01 | 2.87e-02 | **2.09e-18** | 3.78e-02 | 4.41e-02 | 2.57e-02 | 1.21e-01 | 8.42e-01 | 3.27e-01 | 2.93e-01 | 8.24e-03 | 7.28e-01 | 5.81e-01 | 8.78e-01 | 2.96e-01 | 2.61e-01 | 4.13e-01 | 5.53e-01 |
|  |  | 3 | Asn | 9.53e-01 | 4.98e-02 | 2.51e-01 | N.T.*^b^* | 3.33e-02 | **1.89e-13** | 5.23e-01 | 8.31e-01 | 4.61e-01 | 1.21e-01 | 8.48e-01 | 5.24e-01 | 9.59e-01 | 6.86e-01 | 4.26e-01 | 2.90e-01 | 8.63e-01 | 2.75e-01 | 2.94e-01 | 9.33e-01 | 7.06e-01 |
|  |  | 3 | Gln | 4.99e-02 | 4.07e-03 | 2.90e-01 | 3.03e-03 | 9.12e-01 | N.T.*^b^* | 1.96e-01 | 7.56e-03 | 1.13e-03 | 8.61e-01 | 1.05e-03 | 1.79e-01 | 2.19e-03 | 9.16e-04 | 4.72e-01 | 4.43e-01 | 1.17e-03 | 1.70e-04 | 8.62e-01 | 2.00e-02 | 3.06e-01 |
|  |  | 3 | Gly | 8.85e-01 | 5.61e-02 | 2.40e-01 | 8.40e-01 | 3.05e-02 | **3.80e-13** | 5.34e-01 | N.T.*^b^* | 5.58e-01 | 1.28e-01 | 9.84e-01 | 5.16e-01 | 9.55e-01 | 7.84e-01 | 4.29e-01 | 2.74e-01 | 9.95e-01 | 4.59e-01 | 2.92e-01 | 9.96e-01 | 6.94e-01 |
|  |  | 3 | Phe | 8.50e-01 | 3.21e-02 | 3.16e-01 | 9.10e-01 | 4.83e-02 | **2.48e-11** | 5.94e-01 | 8.73e-01 | 3.08e-01 | 1.92e-01 | 6.03e-01 | 8.07e-01 | 7.91e-01 | 5.70e-01 | N.T.*^b^* | 3.85e-01 | 8.41e-01 | 3.26e-01 | 4.42e-01 | 5.59e-01 | 9.97e-01 |
|  |  | 3 | Pro | 6.10e-01 | 4.05e-02 | 3.79e-01 | 8.74e-01 | 6.12e-02 | **3.02e-11** | 6.17e-01 | 7.38e-01 | 3.76e-01 | 2.43e-01 | 6.17e-01 | 7.68e-01 | 7.69e-01 | 4.66e-01 | 6.19e-01 | N.T.*^b^* | 7.47e-01 | 2.06e-01 | 4.09e-01 | 6.17e-01 | 9.80e-01 |
|  |  | 3 | Ser | 8.94e-01 | 4.72e-02 | 2.46e-01 | 8.38e-01 | 3.34e-02 | **1.22e-13** | 5.27e-01 | 8.89e-01 | 5.58e-01 | 1.21e-01 | 9.76e-01 | 5.00e-01 | 9.64e-01 | 7.63e-01 | 4.18e-01 | 2.73e-01 | N.T.*^b^* | 4.09e-01 | 2.90e-01 | 9.99e-01 | 6.81e-01 |
| *PAH* | rs17450273 | |  |  |  |  |  |  |  |  |  |  |  |  |  |  |  |  |  |  |  |  |  |  |
|  |  | 1 |  | 1.97e-01 | 6.73e-01 | 2.06e-02 | 7.23e-02 | 2.38e-01 | 5.36e-02 | 6.01e-01 | 5.89e-02 | 4.25e-01 | 5.83e-01 | 2.62e-02 | 9.61e-01 | 8.77e-01 | 1.80e-01 | **6.60e-10** | 4.54e-01 | 3.33e-02 | 3.33e-01 | 7.52e-01 | 4.73e-01 | 9.37e-01 |
|  |  | 2 |  | 4.58e-01 | 6.36e-01 | 2.77e-02 | 1.27e-01 | 6.80e-01 | 3.69e-01 | 3.60e-01 | 8.15e-01 | 6.51e-01 | 7.75e-01 | 4.72e-05 | 1.58e-01 | 8.31e-03 | 2.66e-01 | **4.00e-23** | 8.98e-01 | 2.42e-01 | 1.26e-03 | 1.58e-01 | 1.89e-01 | 6.27e-01 |
|  |  | 3 | Asn | 4.53e-01 | 5.06e-01 | 7.51e-02 | N.T.*^b^* | 5.23e-01 | 2.35e-01 | 6.13e-01 | 1.75e-01 | 7.54e-02 | 9.29e-01 | 1.82e-01 | 7.21e-01 | 4.46e-01 | 5.97e-01 | **3.87e-13** | 7.54e-01 | 1.41e-01 | 7.66e-01 | 8.28e-01 | 9.61e-01 | 7.65e-01 |
|  |  | 3 | Gln | 3.73e-01 | 5.41e-01 | 6.73e-02 | 2.51e-01 | 5.51e-01 | N.T.*^b^* | 4.92e-01 | 2.14e-01 | 1.16e-01 | 9.34e-01 | 1.36e-01 | 6.49e-01 | 4.61e-01 | 6.15e-01 | **1.05e-11** | 7.60e-01 | 1.32e-01 | 8.60e-01 | 9.79e-01 | 8.85e-01 | 7.80e-01 |
|  |  | 3 | Gly | 2.76e-01 | 7.03e-01 | 4.23e-02 | 1.70e-01 | 4.45e-01 | 1.91e-01 | 4.51e-01 | N.T.*^b^* | 4.04e-01 | 5.80e-01 | 8.10e-02 | 9.08e-01 | 7.77e-01 | 4.57e-01 | **8.06e-10** | 5.97e-01 | 1.70e-01 | 6.83e-01 | 7.79e-01 | 5.62e-01 | 8.67e-01 |
|  |  | 3 | Phe | 8.89e-05 | 3.04e-01 | 7.07e-04 | 5.21e-05 | 1.82e-02 | 5.98e-04 | 1.58e-01 | 6.93e-02 | 4.90e-02 | 1.79e-05 | **1.91e-09** | 1.28e-05 | 1.87e-02 | 1.55e-03 | N.T.*^b^* | 8.73e-03 | 1.87e-03 | 1.23e-02 | 5.02e-04 | 1.37e-07 | 2.35e-04 |
|  |  | 3 | Pro | 2.50e-01 | 6.65e-01 | 2.72e-02 | 9.39e-02 | 3.36e-01 | 8.12e-02 | 6.95e-01 | 8.66e-02 | 3.19e-01 | 8.17e-01 | 4.48e-02 | 8.63e-01 | 9.22e-01 | 3.12e-01 | **2.52e-11** | N.T.*^b^* | 3.97e-02 | 5.00e-01 | 8.85e-01 | 6.48e-01 | 8.35e-01 |
|  |  | 3 | Ser | 3.30e-01 | 3.00e-01 | 5.36e-02 | 3.22e-01 | 4.38e-01 | 2.80e-01 | 4.94e-01 | 4.05e-01 | 2.62e-01 | 7.86e-01 | 1.70e-01 | 8.11e-01 | 6.05e-01 | 5.98e-01 | **7.18e-11** | 6.99e-01 | N.T.*^b^* | 8.48e-01 | 8.71e-01 | 8.17e-01 | 8.95e-01 |
| *ASPG* | rs1744297 |  |  |  |  |  |  |  |  |  |  |  |  |  |  |  |  |  |  |  |  |  |  |  |
|  |  | 1 |  | 5.18e-01 | 4.47e-01 | 1.20e-01 | **1.30e-51** | 6.80e-01 | 5.57e-01 | 2.76e-01 | 4.70e-01 | 8.26e-01 | 9.66e-01 | 3.17e-01 | 9.58e-01 | 7.35e-01 | 5.83e-01 | 7.77e-01 | 7.11e-01 | 3.56e-01 | 2.98e-01 | 2.44e-01 | 7.60e-01 | 4.96e-01 |
|  |  | 2 |  | 5.78e-01 | 7.04e-01 | 6.08e-01 | **3.30e-107** | 3.16e-01 | 8.74e-01 | 4.40e-01 | 8.84e-01 | 3.43e-05 | 8.81e-01 | 8.88e-03 | 2.89e-01 | 2.83e-01 | 4.70e-02 | 6.64e-02 | 3.88e-01 | 1.37e-02 | 4.88e-06 | 1.76e-01 | 5.60e-01 | 4.64e-01 |
|  |  | 3 | Asn | **2.04e-08** | 8.47e-04 | 1.85e-05 | N.T.*^b^* | 1.87e-06 | **1.22e-11** | 3.06e-01 | 2.66e-04 | **1.34e-12** | 8.47e-05 | **9.09e-24** | 9.70e-05 | **1.61e-14** | **1.08e-12** | 4.56e-08 | 4.63e-06 | **1.20e-11** | **8.43e-27** | **1.62e-08** | **8.61e-11** | 3.33e-05 |
|  |  | 3 | Gln | 6.19e-01 | 3.94e-01 | 1.48e-01 | **8.51e-63** | 5.34e-01 | N.T.*^b^* | 3.03e-01 | 5.76e-01 | 9.98e-01 | 9.10e-01 | 4.10e-01 | 8.13e-01 | 5.17e-01 | 3.82e-01 | 6.68e-01 | 5.79e-01 | 4.59e-01 | 3.80e-01 | 1.95e-01 | 5.95e-01 | 4.07e-01 |
|  |  | 3 | Gly | 6.04e-01 | 4.36e-01 | 1.48e-01 | **7.90e-56** | 5.62e-01 | 7.36e-01 | 3.18e-01 | N.T.*^b^* | 8.48e-01 | 9.70e-01 | 4.05e-01 | 9.56e-01 | 6.07e-01 | 4.36e-01 | 7.78e-01 | 6.24e-01 | 5.45e-01 | 3.88e-01 | 2.37e-01 | 7.05e-01 | 5.05e-01 |
|  |  | 3 | Phe | 4.20e-01 | 4.73e-01 | 1.04e-01 | **1.53e-60** | 7.14e-01 | 5.00e-01 | 2.87e-01 | 4.70e-01 | 7.08e-01 | 8.28e-01 | 1.88e-01 | 8.96e-01 | 7.97e-01 | 6.24e-01 | N.T.*^b^* | 7.61e-01 | 3.30e-01 | 2.50e-01 | 2.47e-01 | 8.57e-01 | 5.32e-01 |
|  |  | 3 | Pro | 3.29e-01 | 4.69e-01 | 8.88e-02 | **2.09e-57** | 7.42e-01 | 4.64e-01 | 2.94e-01 | 4.29e-01 | 7.46e-01 | 8.20e-01 | 2.21e-01 | 9.34e-01 | 8.09e-01 | 6.52e-01 | 8.57e-01 | N.T.*^b^* | 3.04e-01 | 1.98e-01 | 2.70e-01 | 8.69e-01 | 5.62e-01 |
|  |  | 3 | Ser | 6.44e-01 | 2.77e-01 | 1.63e-01 | **2.69e-62** | 5.63e-01 | 8.28e-01 | 3.12e-01 | 7.65e-01 | 9.40e-01 | 9.33e-01 | 5.04e-01 | 8.36e-01 | 5.27e-01 | 3.59e-01 | 6.78e-01 | 5.72e-01 | N.T.*^b^* | 5.05e-01 | 2.16e-01 | 5.84e-01 | 4.25e-01 |
| *PRODH* | rs2238732 |  |  |  |  |  |  |  |  |  |  |  |  |  |  |  |  |  |  |  |  |  |  |  |
|  |  | 1 |  | 4.08e-01 | 2.97e-01 | 3.11e-02 | 9.95e-01 | 7.58e-01 | 8.02e-01 | 3.10e-01 | 1.42e-01 | 2.11e-01 | 2.16e-01 | 2.67e-01 | 1.20e-01 | 9.44e-01 | 3.72e-01 | 3.41e-01 | **5.96e-40** | 3.55e-01 | 7.75e-01 | 4.37e-01 | 7.44e-01 | 8.44e-02 |
|  |  | 2 |  | **7.10e-11** | 4.62e-01 | 3.25e-02 | 6.52e-01 | 1.13e-02 | 8.69e-01 | 6.05e-01 | 4.20e-01 | 4.27e-01 | 1.25e-03 | 3.07e-02 | 2.19e-01 | 5.58e-01 | 2.99e-01 | 4.38e-01 | **9.88e-70** | 3.35e-01 | 6.47e-04 | 1.71e-01 | 7.66e-01 | 6.98e-01 |
|  |  | 3 | Asn | 3.63e-01 | 2.91e-01 | 1.96e-02 | N.T.*^b^* | 7.46e-01 | 7.82e-01 | 3.10e-01 | 1.22e-01 | 1.59e-01 | 1.95e-01 | 1.64e-01 | 1.05e-01 | 9.35e-01 | 3.25e-01 | 3.07e-01 | **1.27e-43** | 3.00e-01 | 7.09e-01 | 4.19e-01 | 7.20e-01 | 7.38e-02 |
|  |  | 3 | Gln | 3.41e-01 | 2.76e-01 | 2.80e-02 | 9.12e-01 | 6.86e-01 | N.T.*^b^* | 3.22e-01 | 1.35e-01 | 1.50e-01 | 1.72e-01 | 2.68e-01 | 8.58e-02 | 8.46e-01 | 2.70e-01 | 2.98e-01 | **1.60e-43** | 3.67e-01 | 6.72e-01 | 4.57e-01 | 6.62e-01 | 6.70e-02 |
|  |  | 3 | Gly | 2.62e-01 | 2.81e-01 | 5.57e-02 | 6.54e-01 | 5.24e-01 | 7.39e-01 | 4.12e-01 | N.T.*^b^* | 1.91e-01 | 2.12e-01 | 4.65e-01 | 1.19e-01 | 6.79e-01 | 1.77e-01 | 3.41e-01 | **1.23e-39** | 9.12e-01 | 4.50e-01 | 4.59e-01 | 6.36e-01 | 9.00e-02 |
|  |  | 3 | Phe | 6.03e-01 | 3.92e-01 | 1.89e-02 | 7.41e-01 | 8.97e-01 | 6.26e-01 | 3.73e-01 | 1.42e-01 | 3.44e-01 | 3.79e-01 | 6.85e-02 | 2.18e-01 | 7.88e-01 | 5.19e-01 | N.T.*^b^* | **1.66e-45** | 2.83e-01 | 9.54e-01 | 1.76e-01 | 7.91e-01 | 1.48e-01 |
|  |  | 3 | Pro | **7.40e-19** | 1.17e-02 | 2.75e-01 | 1.60e-04 | 2.68e-04 | 1.86e-04 | 5.23e-03 | 5.72e-01 | 3.11e-06 | **1.24e-14** | 4.53e-05 | **7.10e-11** | 9.95e-05 | **5.48e-09** | 5.99e-07 | N.T.*^b^* | 5.27e-02 | **1.01e-08** | 2.92e-02 | **5.32e-10** | **5.87e-14** |
|  |  | 3 | Ser | 2.93e-01 | 1.67e-01 | 4.43e-02 | 6.57e-01 | 6.37e-01 | 8.76e-01 | 3.49e-01 | 2.37e-01 | 1.57e-01 | 1.68e-01 | 4.30e-01 | 8.26e-02 | 7.24e-01 | 1.98e-01 | 2.75e-01 | **8.82e-41** | N.T.*^b^* | 3.86e-01 | 4.78e-01 | 5.69e-01 | 6.39e-02 |
| *SLC7A2* | rs7838611 |  |  |  |  |  |  |  |  |  |  |  |  |  |  |  |  |  |  |  |  |  |  |  |
|  |  | 1 |  | 5.33e-01 | 8.66e-01 | 1.16e-07 | 6.91e-01 | 2.95e-01 | 8.85e-01 | 5.74e-01 | 8.63e-02 | 6.89e-01 | 3.29e-01 | 9.91e-01 | 7.85e-01 | 3.17e-02 | 2.17e-05 | 6.42e-01 | 3.23e-01 | 4.89e-01 | 9.25e-01 | 1.57e-01 | 5.02e-01 | 6.10e-01 |
|  |  | 2 |  | 5.06e-01 | 3.30e-01 | **1.62e-15** | 3.89e-02 | 1.80e-04 | 1.87e-03 | 2.60e-01 | 2.39e-01 | 4.77e-01 | 5.88e-01 | 4.11e-01 | 3.25e-01 | 1.05e-01 | **7.61e-14** | 5.78e-01 | 6.60e-01 | 9.76e-01 | 6.33e-02 | 3.04e-01 | 4.20e-01 | 5.24e-01 |
|  |  | 3 | Asn | 3.79e-01 | 9.16e-01 | **3.61e-09** | N.T.*^b^* | 2.23e-01 | 7.09e-01 | 5.78e-01 | 5.41e-02 | 7.96e-01 | 2.55e-01 | 7.88e-01 | 6.97e-01 | 9.69e-03 | 1.06e-06 | 5.23e-01 | 2.47e-01 | 3.31e-01 | 6.67e-01 | 1.08e-01 | 3.71e-01 | 5.35e-01 |
|  |  | 3 | Gln | 5.26e-01 | 8.44e-01 | **2.87e-08** | 6.04e-01 | 2.87e-01 | N.T.*^b^* | 5.90e-01 | 7.26e-02 | 6.18e-01 | 3.21e-01 | 9.16e-01 | 8.10e-01 | 2.14e-02 | 2.91e-06 | 6.57e-01 | 3.15e-01 | 4.98e-01 | 9.83e-01 | 1.56e-01 | 5.09e-01 | 6.24e-01 |
|  |  | 3 | Gly | 7.42e-01 | 8.29e-01 | 4.04e-07 | 3.45e-01 | 4.81e-01 | 5.90e-01 | 7.47e-01 | N.T.*^b^* | 6.35e-01 | 3.30e-01 | 5.99e-01 | 7.90e-01 | 7.21e-02 | 8.34e-05 | 6.42e-01 | 4.58e-01 | 7.29e-01 | 6.54e-01 | 1.67e-01 | 6.16e-01 | 5.87e-01 |
|  |  | 3 | Phe | 6.07e-01 | 7.77e-01 | 1.20e-07 | 5.59e-01 | 3.23e-01 | 9.63e-01 | 5.27e-01 | 8.65e-02 | 5.05e-01 | 3.82e-01 | 7.68e-01 | 9.91e-01 | 3.47e-02 | 1.40e-05 | N.T.*^b^* | 3.64e-01 | 5.39e-01 | 9.74e-01 | 1.75e-01 | 6.17e-01 | 7.49e-01 |
|  |  | 3 | Pro | 8.86e-01 | 7.72e-01 | 1.94e-07 | 4.78e-01 | 4.08e-01 | 8.79e-01 | 4.85e-01 | 1.15e-01 | 4.95e-01 | 5.48e-01 | 6.59e-01 | 9.32e-01 | 5.28e-02 | 2.52e-05 | 8.62e-01 | N.T.*^b^* | 6.40e-01 | 7.40e-01 | 2.21e-01 | 7.85e-01 | 9.06e-01 |
|  |  | 3 | Ser | 6.06e-01 | 6.94e-01 | 1.37e-07 | 4.36e-01 | 3.40e-01 | 8.82e-01 | 6.19e-01 | 1.05e-01 | 6.02e-01 | 3.66e-01 | 7.23e-01 | 8.69e-01 | 4.13e-02 | 1.49e-05 | 7.09e-01 | 3.74e-01 | N.T.*^b^* | 7.66e-01 | 1.68e-01 | 6.03e-01 | 6.66e-01 |
| *PKD1L2* | rs8059153 |  |  |  |  |  |  |  |  |  |  |  |  |  |  |  |  |  |  |  |  |  |  |  |
|  |  | 1 |  | 1.18e-01 | 4.54e-01 | 1.75e-01 | 2.79e-02 | 3.74e-01 | 4.49e-03 | 3.98e-01 | 9.08e-07 | 4.55e-02 | 1.56e-02 | 5.83e-03 | 1.88e-02 | 1.04e-01 | 1.52e-02 | 1.80e-01 | 1.67e-02 | 4.79e-01 | 3.75e-02 | 9.71e-01 | 1.06e-02 | 1.34e-01 |
|  |  | 2 |  | 5.06e-01 | 4.08e-01 | 4.87e-01 | 6.77e-01 | 3.46e-01 | 6.86e-01 | 2.09e-01 | **1.46e-08** | 3.31e-01 | 4.83e-01 | 5.36e-01 | 2.60e-01 | 1.96e-01 | 1.35e-01 | 6.60e-01 | 2.23e-01 | 1.50e-04 | 8.54e-01 | 5.17e-02 | 1.51e-01 | 2.11e-01 |
|  |  | 3 | Asn | 4.45e-01 | 7.08e-01 | 5.55e-01 | N.T.*^b^* | 7.45e-01 | 3.88e-02 | 4.19e-01 | 7.52e-06 | 2.44e-01 | 5.17e-02 | 7.87e-02 | 6.37e-02 | 4.93e-01 | 9.93e-02 | 4.85e-01 | 6.02e-02 | 7.01e-01 | 3.79e-01 | 5.21e-01 | 5.91e-02 | 3.09e-01 |
|  |  | 3 | Gln | 4.33e-01 | 7.13e-01 | 6.07e-01 | 2.34e-01 | 9.16e-01 | N.T.*^b^* | 5.96e-01 | 2.55e-05 | 2.88e-01 | 7.13e-02 | 9.03e-02 | 1.09e-01 | 6.42e-01 | 1.64e-01 | 4.35e-01 | 8.38e-02 | 5.81e-01 | 2.90e-01 | 6.24e-01 | 7.26e-02 | 3.56e-01 |
|  |  | 3 | Gly | 4.85e-01 | 5.14e-01 | 6.38e-01 | 3.61e-01 | 9.36e-01 | 2.64e-01 | 8.83e-01 | N.T.*^b^* | 6.06e-02 | 1.50e-02 | 1.35e-01 | 1.89e-02 | 5.86e-01 | 2.70e-01 | 1.58e-01 | 9.09e-02 | 1.70e-02 | 4.77e-01 | 9.45e-01 | 3.12e-02 | 1.07e-01 |
|  |  | 3 | Phe | 2.38e-01 | 6.35e-01 | 2.28e-01 | 4.74e-02 | 4.76e-01 | 7.86e-03 | 2.98e-01 | 8.58e-07 | 1.02e-01 | 4.18e-02 | 1.47e-02 | 5.71e-02 | 2.16e-01 | 3.41e-02 | N.T.*^b^* | 3.31e-02 | 5.81e-01 | 6.20e-02 | 5.10e-01 | 2.38e-02 | 3.51e-01 |
|  |  | 3 | Pro | 6.97e-01 | 6.18e-01 | 4.05e-01 | 9.27e-02 | 7.14e-01 | 2.23e-02 | 2.45e-01 | 4.27e-06 | 1.37e-01 | 1.25e-01 | 4.54e-02 | 1.09e-01 | 3.23e-01 | 9.06e-02 | 4.74e-01 | N.T.*^b^* | 8.24e-01 | 1.92e-01 | 6.10e-01 | 7.98e-02 | 5.60e-01 |
|  |  | 3 | Ser | 1.37e-01 | 5.65e-01 | 1.97e-01 | 2.69e-02 | 3.94e-01 | 4.24e-03 | 4.50e-01 | 7.76e-08 | 4.86e-02 | 1.69e-02 | 6.08e-03 | 2.25e-02 | 1.35e-01 | 1.98e-02 | 1.88e-01 | 1.91e-02 | N.T.*^b^* | 4.16e-02 | 9.98e-01 | 1.16e-02 | 1.52e-01 |

The significant associations are highlighted as grey background and bold font. In GWAS-2, conditional PFAA analysis using multiple linear regression models listed in Table S2 was performed. In GWAS-3, each PFAA was adjusted by one of the six PFAAs (asparagine, glutamine, glycine, phenylalanine, proline, and serine). *a*; single nucleotide polymorphism, *b*; Not tested
