# Supplementary material for: Genetic basis for plasma amino acid concentrations based on absolute quantification: a genome-wide association study in the Japanese population
Source: Eur J Hum Genet. 2019 Jan 18;27(4):621–30. doi: 10.1038/s41431-018-0296-y (PMC6460579; doi:10.1038/s41431-018-0296-y)
Supplement: Supplementary file 5 — Supplementary table S5 [file 41431_2018_296_MOESM5_ESM.docx]

Supplementary table S5. Candidates of contributing genetic variation for plasma amino acid levels and prediction of functional impact of these variations.

| Gene | SNPs*^a^*  (rs ID) | Chr*^b^* | Position | Tag SNPs | *r*^2^ | Ref./Var | Amino acid | SIFT Prediction  (ref. 25) | PolyPhen Prediction  (ref. 26) |
| --- | --- | --- | --- | --- | --- | --- | --- | --- | --- |
| ***Identified by GWA studies*** | | |  |  |  |  |  |  |  |
| *SLC7A2* | rs56335308 | 8 | 17419461 |  |  | G/A | Val/Met | tolerated (0.09) | benign (0.007-0.146) |
| *GLS2* | rs2657879 | 12 | 56865338 |  |  | A/G | Leu/Pro | tolerated (0.31) | benign (0) |
| *PRODH* | rs450046 | 22 | 18901004 |  |  | C/T | Gln/Arg | tolerated (0.39-0.42) | benign (0) |
| ***Identified by in-depth LD analysis*** | | |  |  |  |  |  |  |  |
| *CPS1* | rs1047891 | 2 | 211540507 | rs715 | 0.839 | C/A | Thr/Asn | tolerated (0.18-0.24) | benign (0.01-0.017) |
| *ASPG* | rs8012505 | 14 | 104571054 | rs1744297 | 0.943 | C/G | Ser/Arg | deleterious (0.02-0.03) | benign (0.022-0.048) |
| *PKD1L2* | rs8054182 | 16 | 81145807 | rs8059153 | 0.989 | C/G | Met/Ile | - | - |
| *PRODH* | rs5747933 | 22 | 18910355 | rs2277834 | 0.939 | G/T | Thr/Asn | tolerated (0.4) | possibly damaging (0.614) |

Impacts were predicted using Ensembl Variant Effect Predictor (VEP) (ref. 24).

Imputation was performed using the genotyping results of 665 samples that were unrelated to those used for the present study.

*a*; single nucleotide polymorphism, *b*; chromosome
